# Nano and Battery Anode: A Review

**DOI:** 10.1186/s11671-021-03631-x

**Published:** 2021-12-11

**Authors:** Hasan Sh. Majdi, Zagir Azgarovich Latipov, Vitaliy Borisov, Nedorezova Olga Yuryevna, Mustafa M. Kadhim, Wanich Suksatan, Ibrahim Hammoud Khlewee, Ehsan Kianfar

**Affiliations:** 1 Department of Chemical Engineering and Petroleum Industries, Al-Mustaqbal University College, Babylon, 51001 Iraq; 2 Elabuga Institute of KFU, Kazan Federal University, Elabuga, Russia; 3 Sechenov First Moscow State Medical University, Moscow, Russia; 4 Department of Legal and Social Sciences, Naberezhnye Chelny Institute, Kazan Federal University, Kazan, Russia; 5 Department of Dentistry, Kut University College, Kut, Wasit 52001 Iraq; 6 Faculty of Nursing, HRH Princess Chulabhorn College of Medical Science, Chulabhorn Royal Academy, Bangkok, 10210 Thailand; 7 Department of Prosthodontics, College of Health and Medical Technololgy, Al-Ayen University, Thi-Qar, Iraq; 8 Department of Chemical Engineering, Arak Branch, Islamic Azad University, Arāk, Iran; 9 Young Researchers and Elite Club, Gachsaran Branch, Islamic Azad University, Gachsaran, Iran; 10 College of Technical Engineering, The Islamic University, Najaf, Iraq; 11 Department of Pharmacy, Osol Aldeen University College, Baghdad, Iraq

**Keywords:** Battery anode, High-capacity anodes, Changing storage mechanism, Capacitive quasi-capacitance, Titanium oxide

## Abstract

Improving the anode properties, including increasing its capacity, is one of the basic necessities to improve battery performance. In this paper, high-capacity anodes with alloy performance are introduced, then the problem of fragmentation of these anodes and its effect during the cyclic life is stated. Then, the effect of reducing the size to the nanoscale in solving the problem of fragmentation and improving the properties is discussed, and finally the various forms of nanomaterials are examined. In this paper, electrode reduction in the anode, which is a nanoscale phenomenon, is described. The negative effects of this phenomenon on alloy anodes are expressed and how to eliminate these negative effects by preparing suitable nanostructures will be discussed. Also, the anodes of the titanium oxide family are introduced and the effects of Nano on the performance improvement of these anodes are expressed, and finally, the quasi-capacitive behavior, which is specific to Nano, will be introduced. Finally, the third type of anodes, exchange anodes, is introduced and their function is expressed. The effect of Nano on the reversibility of these anodes is mentioned. The advantages of nanotechnology for these electrodes are described. In this paper, it is found that nanotechnology, in addition to the common effects such as reducing the penetration distance and modulating the stress, also creates other interesting effects in this type of anode, such as capacitive quasi-capacitance, changing storage mechanism and lower volume change.

## Introduction

**Fig. 1:**
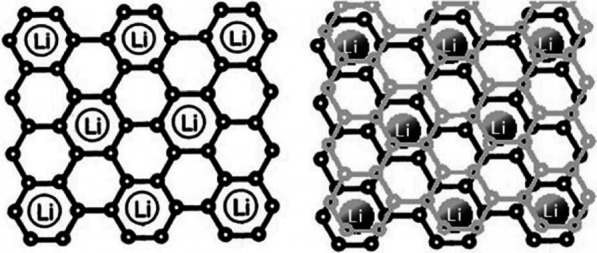
Shows how lithium is stored in graphite. For every 6 carbon atoms, 1 lithium atom is stored [12]

**Fig. 2:**
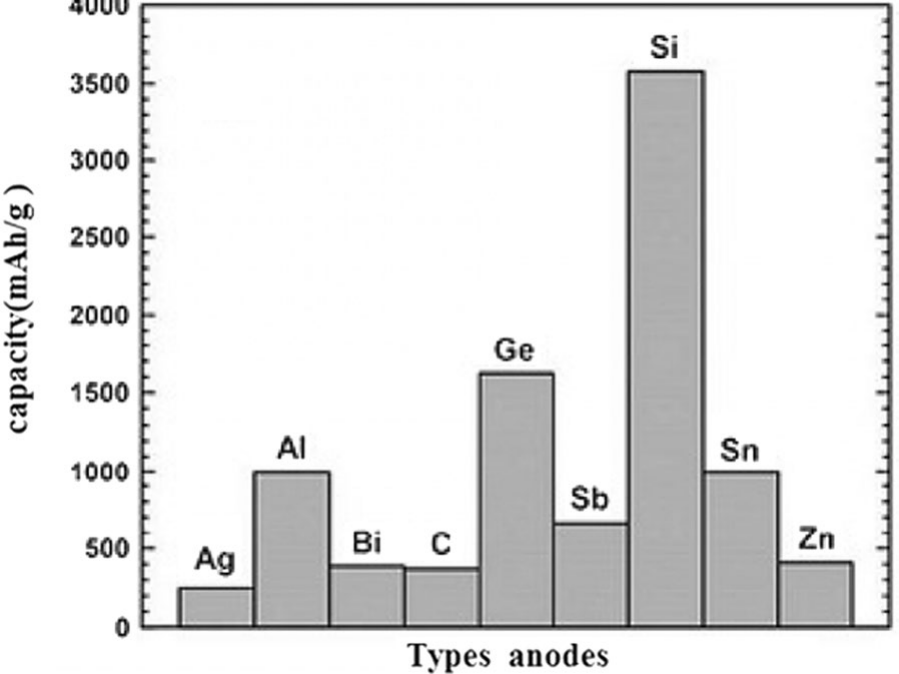
Types of anodes with capacity [13–15]

Graphite is a carbon material with a layered structure in which the distance between the layers is about 35.3 Å, in which there is a suitable space between the layers for the placement of lithium atoms [1–4]. During charging, lithium ions are reduced in the anode and converted to lithium atoms, which are placed between the graphite layers. After the arrival of lithium, the distance between the plates reaches 3.5 Å [5–10]. During discharge, the lithium atoms are oxidized to lithium ions and transported through the electrolyte to the cathode. Due to the insertion of lithium atoms in graphite (at the time of charging), these materials are called intercalation anodes [6–14]. According to Fig. 1 in graphite, a maximum of one lithium atom can be stored for every 6 carbon atoms [5]. Because capacity is directly related to the amount of lithium stored, graphite has a lower capacity than lithium metal anodes, but as mentioned earlier, it is used as a commercial anode because it does not have dendritic growth problems. Note that in this article and future articles, the anode and cathode means the active substance in the anode and cathode [6–8]. Due to the low capacity of graphite, anodes with high capacity are required [15–18]. A group of anodes that can store large amounts of lithium atoms are alloy-type anodes made of metal or semiconductors. The function of these anodes is to form an alloy with a metal or semiconductor, thereby storing the lithium atom [19–21]. In this type of material, compared to graphite, where only one lithium atom is stored for every 6 carbon atoms, several lithium atoms can be stored for each metal atom [9–11]. The most important of these anodes are silicon, and antimony. For silicon the capacity is about 4000 mAh/g and for tin the mentioned capacity is 900 mAh/g compared to graphite which has a capacity of about 350 mAh/g. According to Fig. 2, among alloy anodes, silicon has the highest volume and weight capacity, is found in abundance in nature, and the entire electronics industry is based on silicon; Thus, as Fig. 2 shows, silicon is the most important of the alloy anodes [12–15]. Therefore, most of the material in this article is about silicon, but the principles mentioned can be generalized to other alloy anodes. Active anode material, Theoretical capacity, Advantages and study results are presented in Table 1.

**Table 1:** Research on active anode material, theoretical capacity, advantages

Active anode material	Theoretical capacity (mAh g^−1^)	Advantages	Common issues	References
**Insertion/de-insertion materials** A. Carbonaceous a. Hard carbons b. CNTS c. Graphene	200–600 1116 780/1116	Good working potential Low cost Good safety	Low coulombic efficiency High voltage hysteresis High irreversible capacity	[3, 22–28]
**Insertion/de-insertion materials** B. Titanium oxides a. LiTi_4_O_5_ b. TiO_2_	175 330	Extreme safety Good cycle life Low cost High power capability	Very low capacity Low energy density	[29]
**Alloy/de-alloy materials** a. Silicon b. Germanium c. Tin d. Antimony e. Tin oxide f. SiO	4212 1624 993 660 790 1600	Higher specific capacities High energy density Good safety	Large irreversible capacity Huge capacity fading Poor cycling	[25, 26, 30–34]
**Conversion materials** a. Metal oxides (Fe_2_O_3_, Fe_3_O_4_, CoO, Co_3_O_4_, MnxOy, Cu_2_O/CuO, NiO, Cr_2_O3, RuO_2_, MoO_2_/MoO_3_ etc.)	500–1200	High capacity High energy Low cost Environmentally compatibility High specific capacity Low operation potential and Low polarization than counter oxides	Low coulumbic efficiency Unstable SEI formation Large potential hysteresis Poor cycle life Poor capacity retention Short cycle life High cost of production	[32, 33, 35–38]
**Conversion materials** b. Metal phoshides/sulfides/nitrides (MXy; M ¼ Fe, Mn, Ni, Cu, Co etc. and X ¼ P, S, N)	500–1800	High capacity High energy Low cost Environmentally compatibility High specific capacity Low operation potential and Low polarization than counter oxides	Low coulumbic efficiency Unstable SEI formation Large potential hysteresis Poor cycle life Poor capacity retention Short cycle life High cost of production	[33, 37, 38]

## Problems of Alloy Anodes

**Fig. 3:**
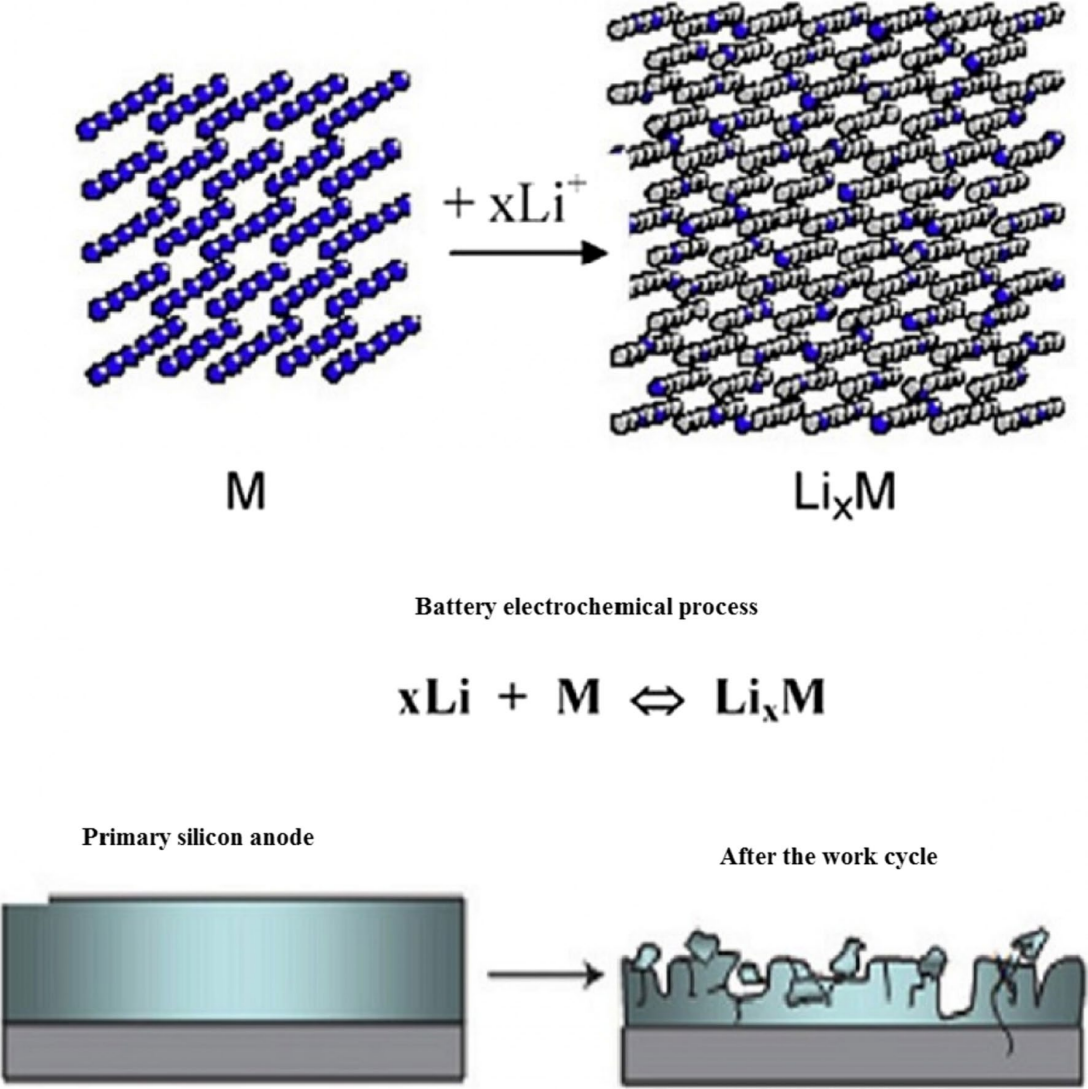
Pulverization and disconnection of its electrical connection [10]

**Fig. 4:**
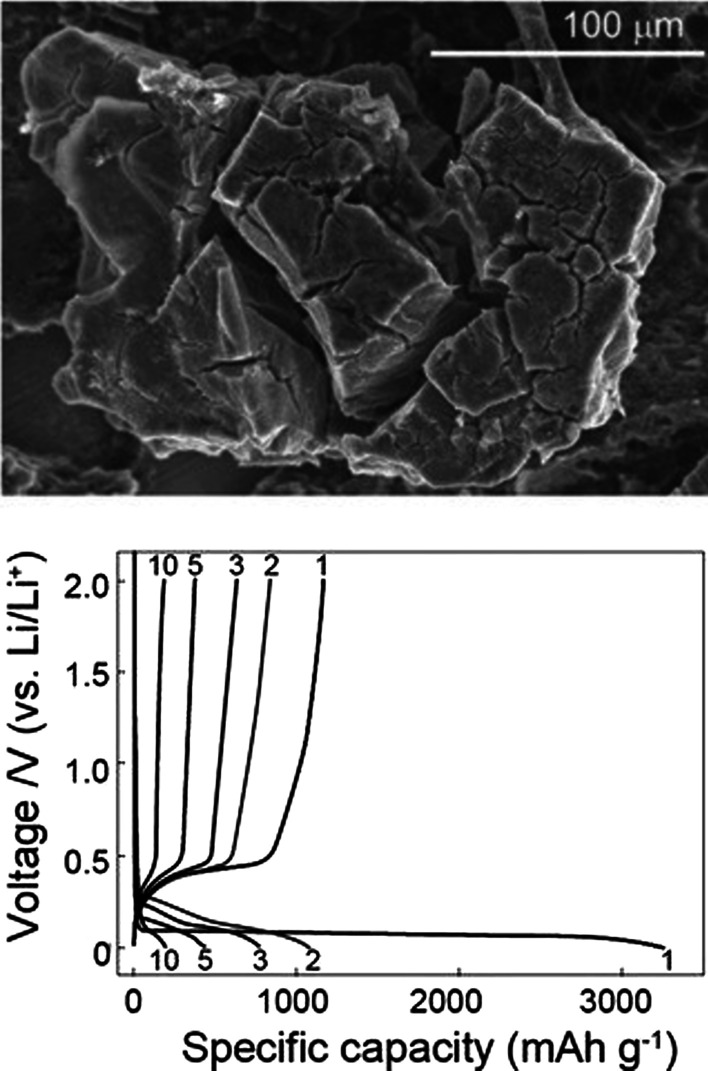
Charging and discharging diagram for 10-micron silicon particles [17]

In these anodes, the storage and release of lithium is accompanied by a large volume change that can reach up to 400% of the initial volume, as shown in Fig. 3. During the work cycle, due to the stresses caused by volume change, the phenomenon of pulverization of active substances occurs [7, 10, 39, 40]. Fragmentation causes the connection between the active material itself, between the active conductive–additive material and between the active-collecting active substance to be cut off [18–20]. When this phenomenon occurs, the active substance is electrically isolated; therefore, electron transfer does not take place to carry out the oxidation reaction. Therefore, a large volume of active ingredients remains unused and do not participate in capacity, and ultimately this causes a sharp drop in capacity during the work cycle [21, 41, 42]. Figure 3 shows the crushing phenomenon. Unfortunately, Fig. 3 does not show the entire structure of the anode electrode. In fact, in a conventional electrode, micron particles of active materials are used along with binders and carbon conductive materials, etc. [43–46]. Which is broken if the electronic connections mentioned above are broken. Figure 4 shows the charge and discharge curves for silicon particles measuring 10 microns. It can be seen that the capacity even at the first discharge is only 800 mAh/g (compared to the initial 4000 charged). In graphite, on the other hand, capacity decreases by only 0.03 per work cycle. These anodes have a higher voltage than graphite (according to the formula stated earlier, the higher the anode voltage, the lower the battery voltage) [10, 21, 39]. For example, silicon has a voltage of 0.3 to 0.4 higher than lithium, while in graphite the voltage is about 0.05 V higher than lithium, but silicon and other alloy anodes have such a high capacity that the voltage does not have a significant effect, and the energy is significantly higher than graphite.

## Nanotechnology Solution

**Fig. 5:**
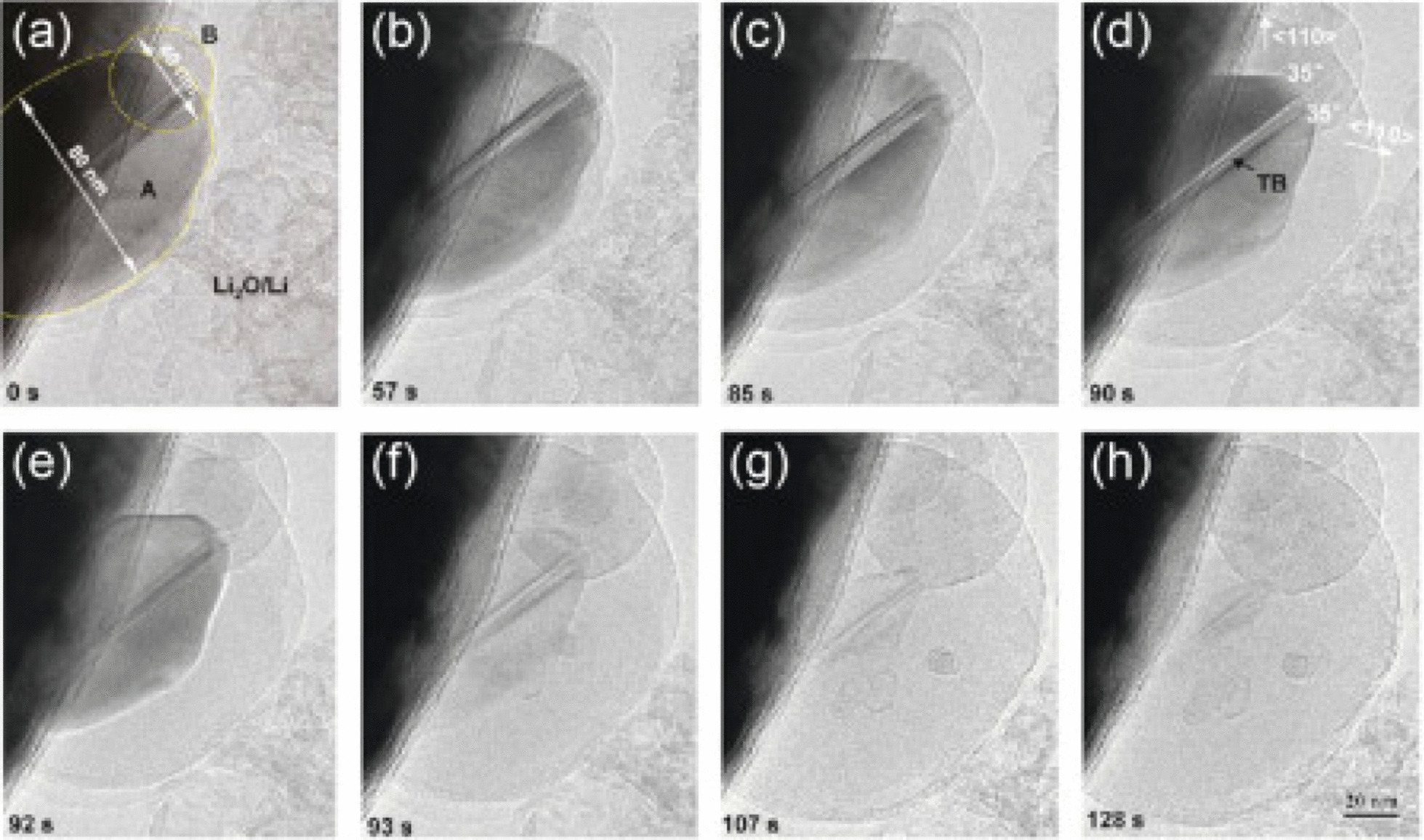
TEM image of silicon nanoparticles during lithium ionization progressed from **a** to **h** it lithium ionization, respectively [19]

**Fig. 6:**
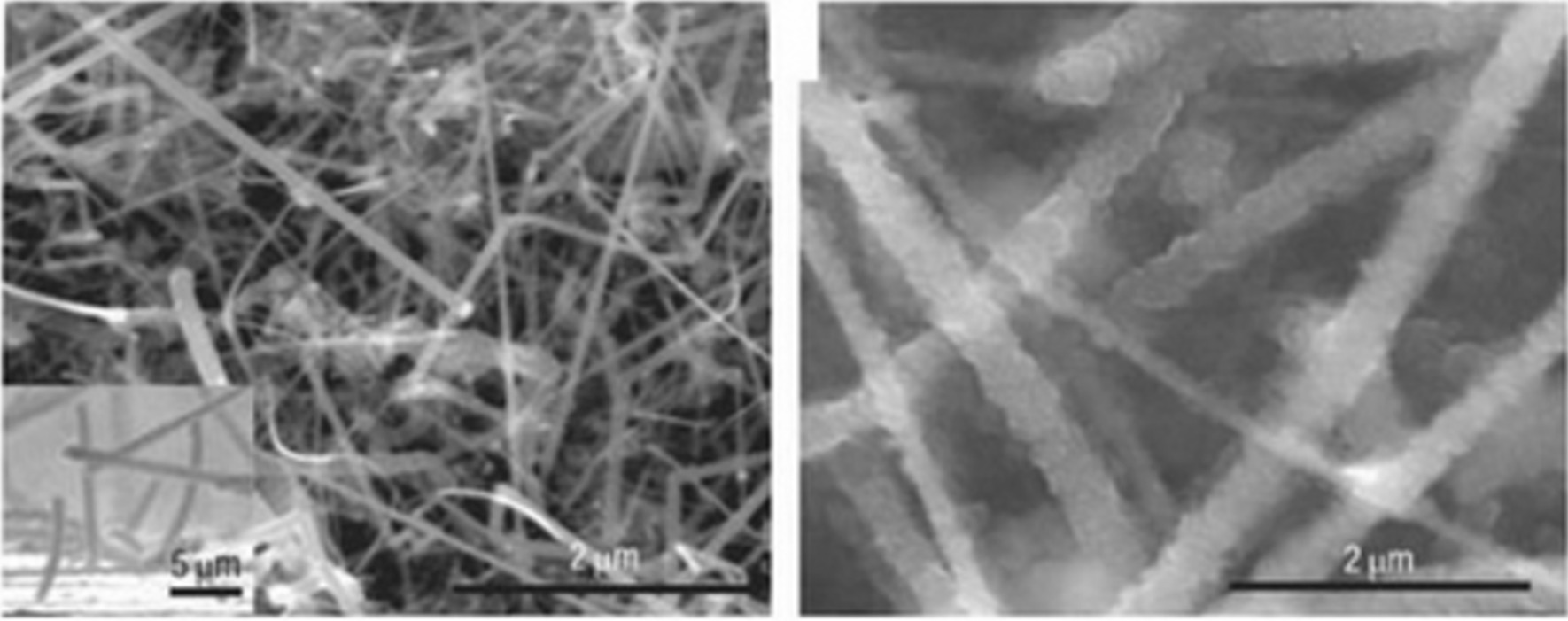
SEM image of silicon nanoparticles during lithium [20]

Battery performance can be improved if the shredding phenomenon can be prevented in some way. Research has shown that when the dimensions of silicon reach the nanometer range (less than 150 nm), the crushing phenomenon no longer occurs [47–50]. Figure 5 shows the TEM image of silicon nanoparticles during lithium ionization. These two particles change volume due to the entry of lithium, but do not break under stress [7, 18, 40]. This shows that in order to use the extraordinary capacity of silicon, we must inevitably go to the nanoscale [51–53]. If nanoparticles are used, the problem of fragmentation is solved, but they are not normally connected to the electron supply. Therefore, for the first time, the researchers used silicon nanowires grown vertically on a current collector as shown in Fig. 6 (SEM image). In this way, the problem of crushing can be solved, because there is enough space between the nanowires to change the volume of each nanowire during the duty cycle without generating extensive stress, the diameter of each nanowire is also less than the critical dimension [19–22, 30, 54–56]. As it is known, after alloying (entry of lithium), the width of the nanowires increased and the side walls became textured, and although there was a large volume change, no fragmentation occurred [57]. In nanowires, electron transfer (communication between the current collector and the active substance) takes place through the length of the nanowires. Since the electron transfer is good, the full capacity of the silicon active material can be used [31, 32, 35–37]. Nanowires have a higher electrolyte bond season than bulk material [41–43]. Due to the fact that the oxidation reaction takes place through the electrode–electrolyte interface, the speed of the reactions also increases. on the other hand, because nanowires have small dimensions compared to the bulk material that ions have to travel longer distances, ion transfer through lateral dimensions is easy. Faster ion transfers and oxidation reactions increase power and even energy, because ion transfer (sometimes in addition to electron transfer) is a potential loss (concentration polarization) of both the anode and cathode electrodes of a lithium battery, this polarization decreases as the penetration distance decreases and the energy density improves [44–46]. Finally, because silicon is a semiconductor, it has less electron conductivity than graphite, which is a metalloid [33, 38, 58].

## Different Nano Morphologies

**Fig. 7:**
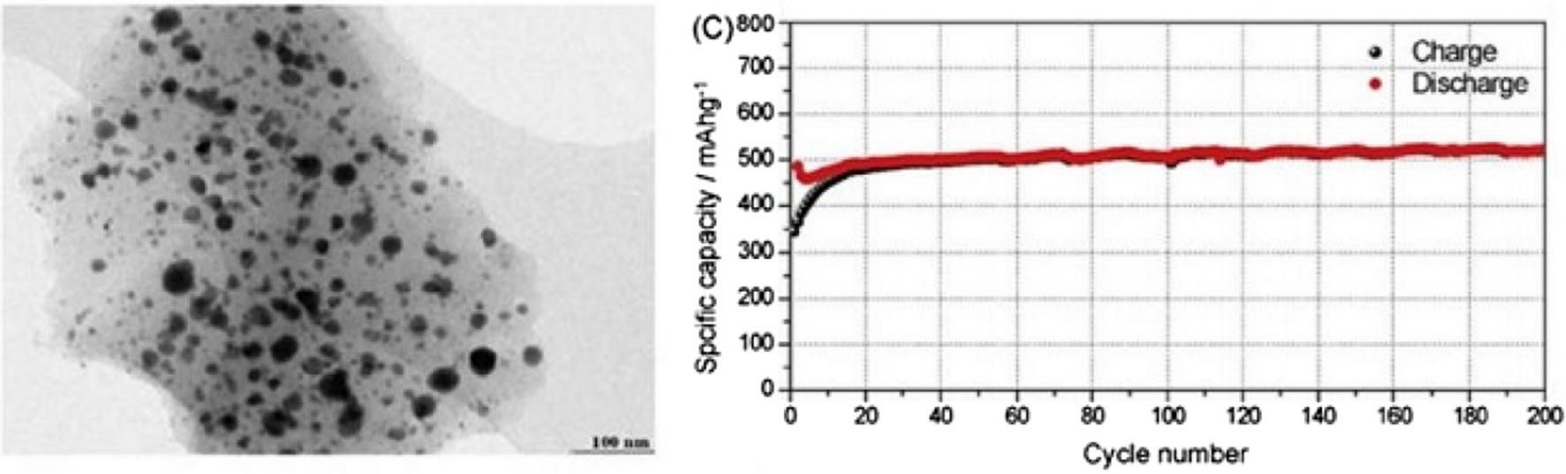
TEM image and life cycle curve of tin nanocomposite in carbon, dark tin nanoparticles are marked [54]

It has been shown that using silicon nanotubes instead of nanowires is more effective. In nanotubes, the necessary space is provided for volume change on both sides of the inner and outer walls [34, 59–61]. In addition, nanotubes are usually thinner than nanowires, so transmitters are better, because silicon is a semiconductor and is also amorphous during the duty cycle due to stresses, it does not conduct well electronically during the duty cycle [47, 48, 54]. As a result, electrons do not flow well in all parts of the silicon. Hybrid nanostructures can be used to solve this problem, for example a silicon nanotube whose core contains conductive materials or vice versa has a conductive coating [62–66]. A comparison between the two categories of uncoated and carbon-coated silicon nanowires has shown that carbon-coated nanowires maintain considerable capacity. Another solution is to use nanocomposite anodes [49–51]. One of the most widely used materials in nanocomposites in the role of stress modulator (buffer) is carbon. For example, carbon nanocomposite in the field of carbon is one of the solutions to the stress problem. Figure 7 shows a Tin–carbon nanocomposite. Tin acts as an active ingredient as an alloy anode. Carbon in this nanocomposite acts as both a buffer and a conductor, and in addition to their various carbon structures, they can store some lithium. As shown in Fig. 7, the tin capacity is less than the theoretical capacity (900 mAh/g) due to the presence of carbon, but has a good cycle life. Maintains well up to 1000 working cycles [52, 67–70].

**Fig. 8:**
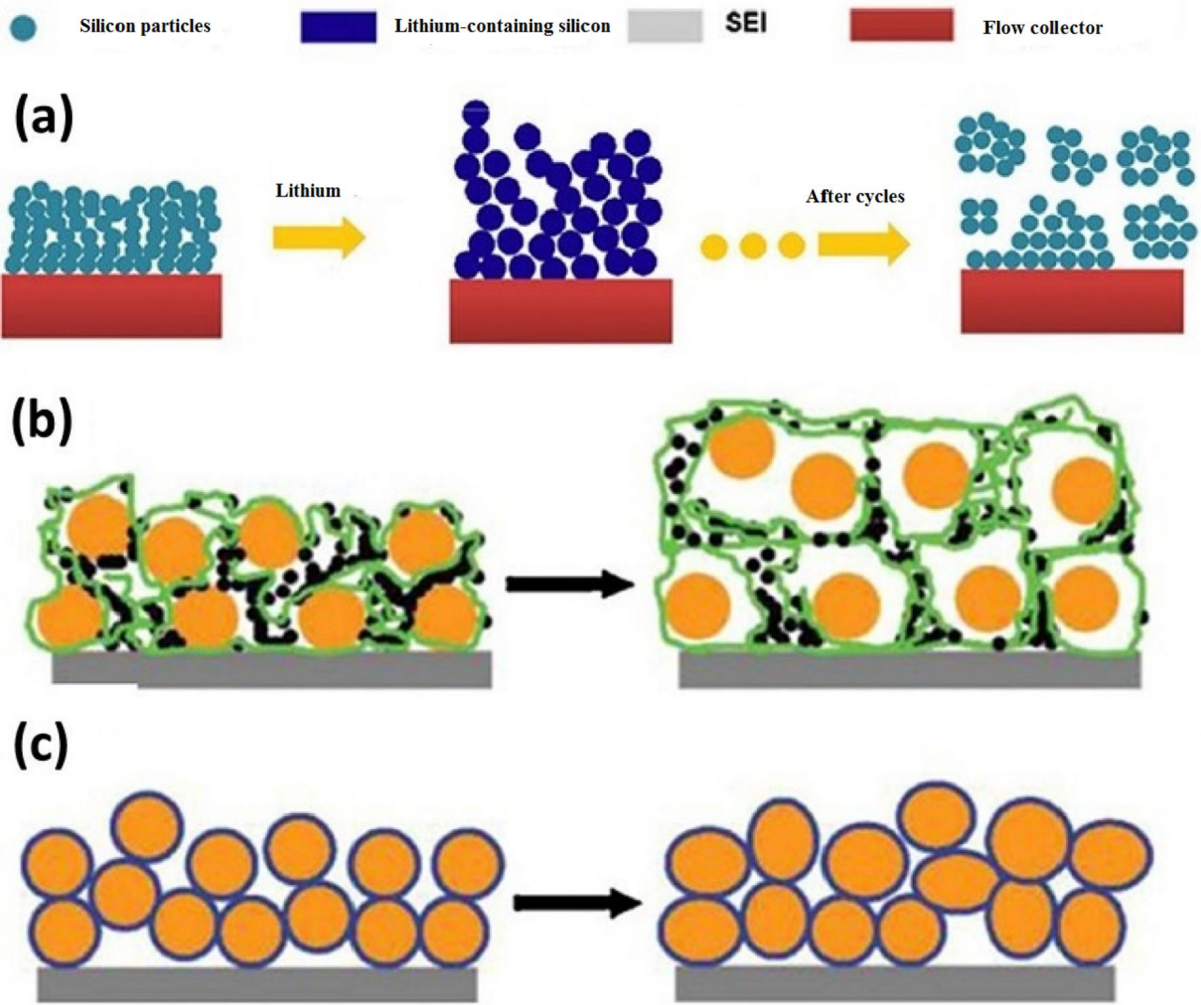
**a** Shows how the electrical relationship of nanoparticles with the current collector is broken, **b** shows another type of disconnection, silicon nanoparticles in orange and carbon in black and PDVF polymer chains are shown in green. **c** Use amorphous silicon adhesive to bond nanoparticles even after deformation [47].

**Fig. 9:**
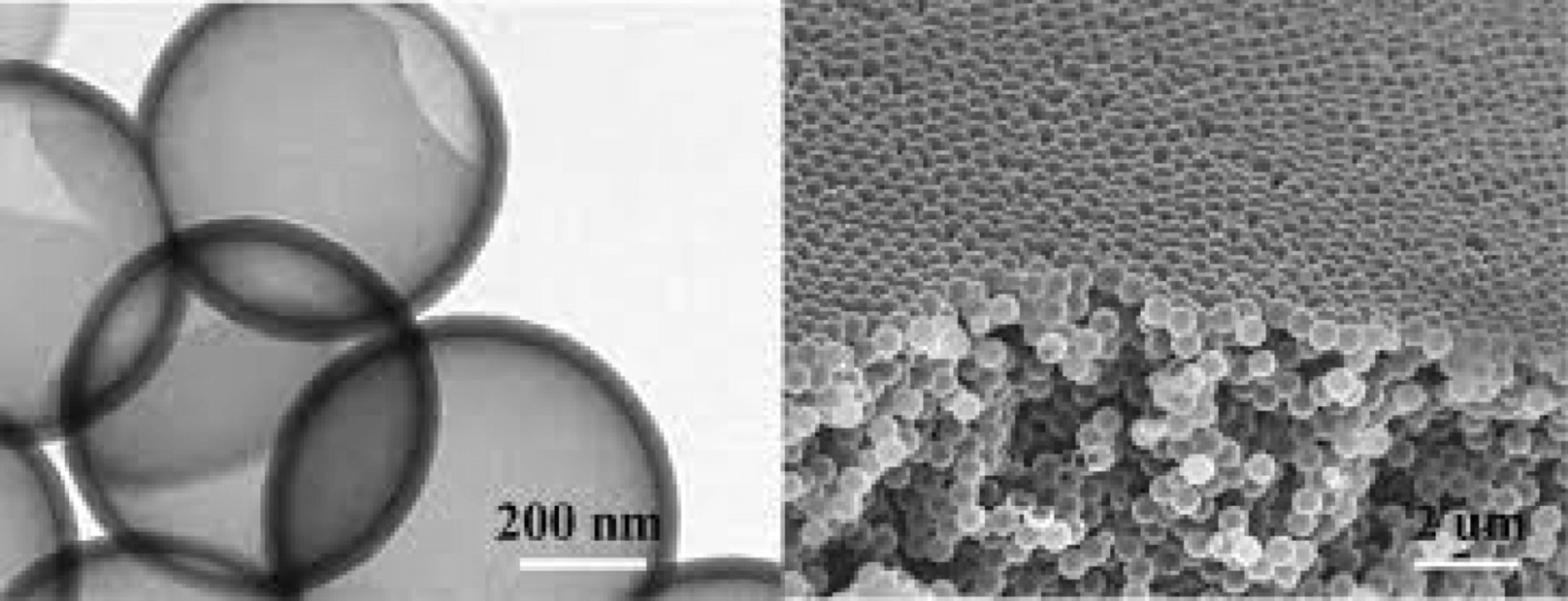
Hollow nanoparticles to solve the volume change problem [48]

The question may arise in the mind of the reader as to why other alloy anodes are being explored, given that silicon has a much higher capacity than other alloy anodes [71–73]. The answer that is given and can be generalized to the whole collection of nanotechnology and battery articles is that because nanomaterials are synthesized in different ways and with different morphologies (shapes) in different ways [74–78]. Each synthesis method is different from the discussion of price, quality, safety, scalability, environmental effects, etc.; for example, metals cannot be prepared with the sol–gel method, which is a simple method [79–81]. Even for a specific material such as silicon, one-dimensional nanomaterials, such as nanofibers, can be produced by electrospinning, which is a mass-produced method, in the form of nanowires by the expensive chemical vapor deposition method, another method for laboratory testing [22, 52–54]. Nanowires can be fabricated by silicon etching. In the latter method, the crystalline direction and doping can be easily controlled, and the effect of different dopants and crystalline directions on lithium storage can be determined [23, 82–88]. Even a nanomaterial with a specific shape and composition can be used in different ways and even in a specific method, different reactants can be used with different temperature conditions, etc., each of which may have different results in terms of price, safety and since the key to commercialization other than investing is to find the right production method by considering the factors listed above, so there is an inseparable link between production and performance in batteries and very good and appropriate articles Available in connection with the synthesis method [30, 31, 56, 57, 89–92]. In addition to one-dimensional nanostructures (nanowires and nanotubes), efforts have been made to use zero-dimensional nanostructures (nanoparticles) (as a good nanoparticle are easier to synthesize than nanowires). The problem with nanoparticles is that on the one hand it is not possible to easily make a connection between the nanoparticles themselves and on the other hand between them and the conductive and collecting material [32, 35, 36]. For example, Fig. 8a shows that the primary nanoparticles (left of the image) increase in volume after absorbing lithium during charging, and after a few cycles, disconnect the electron connection when it returns to its original state without lithium [3, 24, 93–95]. In the usual method of preparing the anode (also the cathode), the powder of the active substance (here silicon) is used together with the conductive carbon (to improve the conductivity) and the PDVF binder (for the bonding of the particles) shown in Fig. 8b. According to Figure b, because the silicon nanoparticles change volume, after returning to another initial state, the electrical connection between the nanoparticles, the carbon conductive material is lost, the capacity is reduced. To solve the above problem in the method shown in Figure c, amorphous silicon, which also has a stress-modulating role, is used as an adhesive to bond silicon nanoparticles so that the electrical connection is no longer interrupted and the capacity will remain [25, 37, 38, 96, 97]. In another method, nanoparticles in the field of polyaniline conductive polymer, which have both a modulating and electron conducting role, have been prepared and observed to have a good cycle life of 1000 while maintaining a capacity of 1600 mAh/g. In comparison, the PVDF binder method loses more than 50% of its capacity in 100 working cycles. Another way to solve the problem is hollow nanostructures. In this method, the necessary empty space is provided during the entry and exit of lithium through a hollow volume [26, 27, 33, 58, 59]. The finite element method shows that in the same volume, the hollow structure undergoes less stress during the work cycle, so they have better resistance to the crushing phenomenon (Fig. 9).

## Electrolyte Decomposition in the Anode

As we know, any substance is stable in a potential range and undergoes a reduction or oxidation process more or less within this range [28, 98, 99]. That is why we can decompose (electrolyze) water to produce hydrogen and oxygen. These cells are the opposite of galvanic cells (batteries), called electrolyte cells. In these cells, unlike the battery, we give energy to force a reaction that is not thermodynamically desirable [60].

**Fig. 10:**
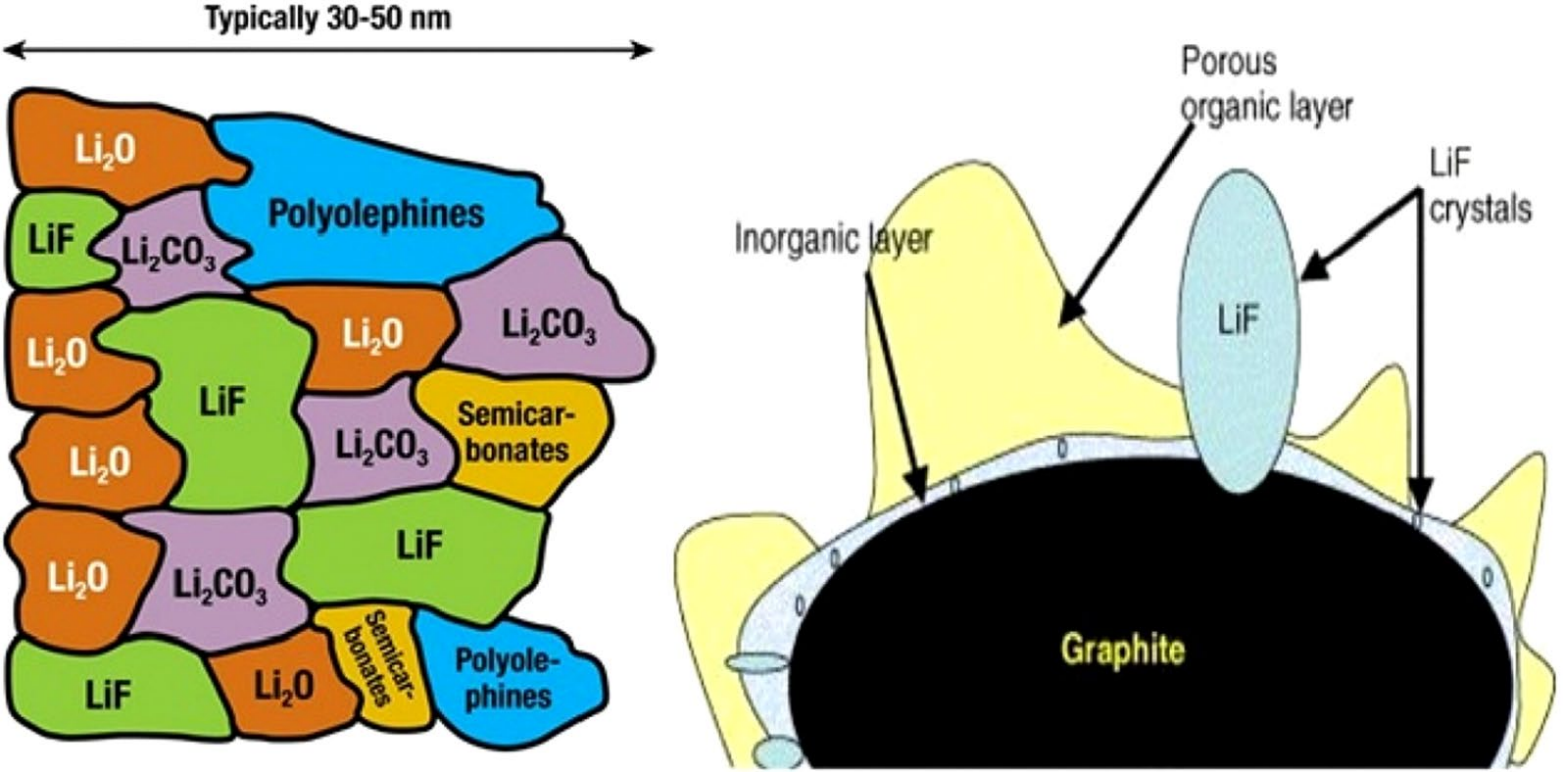
Schematic of SEI formation and composition of this layer [66]

When charging the battery, just like decomposing water, we give energy to the battery through the charger to reverse the reaction that took place in the battery and return the battery to its pre-discharged state [100–104]. The organic electrolyte used in lithium-ion batteries (such as water electrolysis) changes as a result of the energy from the charger. As mentioned, in a lithium-ion battery, at the negative pole (graphite anode), lithium-ion reduction occurs during charging. Due to the fact that the tendency to electrolyte reduction is thermodynamically higher than lithium ion, so electrolyte reduction is done instead of lithium ion reduction. This causes a solid layer to form on the graphite surface. This solid layer is called SEI (solid electrolyte interface). The composition of this layer is complex and a mixture of several chemicals. Figure 10 shows a schematic of this layer. As can be seen from the figure, the composition of this substance contains lithium ions and carbon; therefore, the formation of this layer is accompanied by a decrease in lithium, which reduces the capacity in the first charge [34, 61]. This layer of thickness is in the nanometer range as shown in Fig. 10. The formation of the SEI layer itself limits the continuation of the electrolyte reduction reaction, because it prevents the electrolyte molecules from reaching the graphite anode surface as a physical barrier. In fact, it acts as a kinetic inhibitor (like the passive layer of aluminum oxide, which prevents oxygen from reaching the lower aluminum and prevents the rest of the aluminum from oxidizing). On the other hand, because it is an electron insulator, it also prevents the electron from reaching the electrolyte [62–65]. Therefore, neither the electron can reach the electrolyte molecule nor the electrolyte molecule can move towards the electron in the anode, both of which cause the electrolyte to regenerate and have a self-limiting reaction. Fortunately, this layer is permeable to lithium ions, and lithium ions can pass through it to the anode surface, capture electrons, and regenerate [105–108]. This layer reduces battery power as it increases the penetration distance of the lithium ion to reach the anode [109–111].

**Fig. 11:**
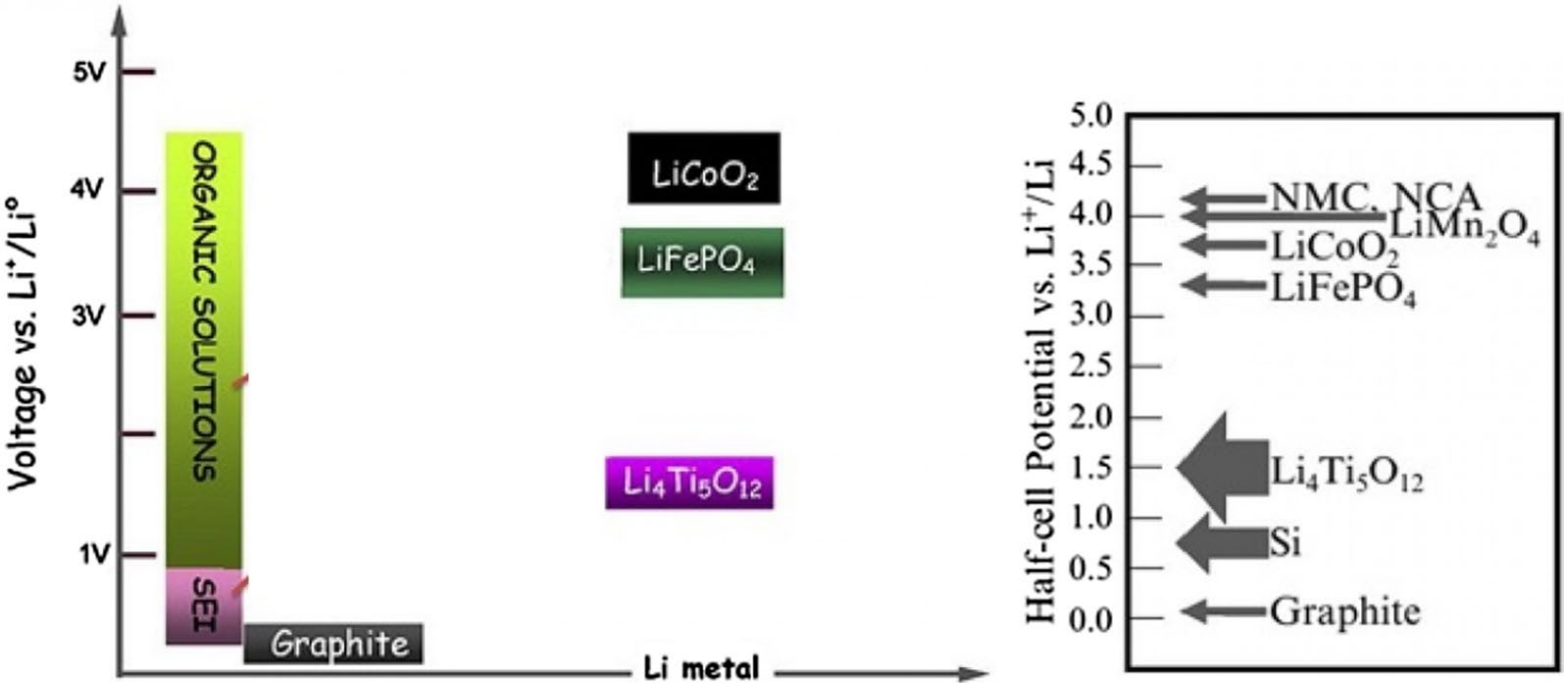
Shows the voltage of common anodes and cathodes and the range of electrolyte stability potential and the potential range of SEI formation [67]

Figure 11 shows the range of electrolyte stability against the potential of anodes and cathodes. If the cathode has a potential higher than the electrolyte stability range, the electrolyte is oxidized at the cathode and during charging, and also if the anode has a lower potential than the stability range, it is regenerated at the anode and during electrolyte charging. Fortunately, as shown in Fig. 11, conventional cathodes do not have the problem of electrolyte instability, but in graphite and silicon anodes there is instability and SEI is formed [66–68].

## SEI Problem in Silicon

**Fig. 12:**
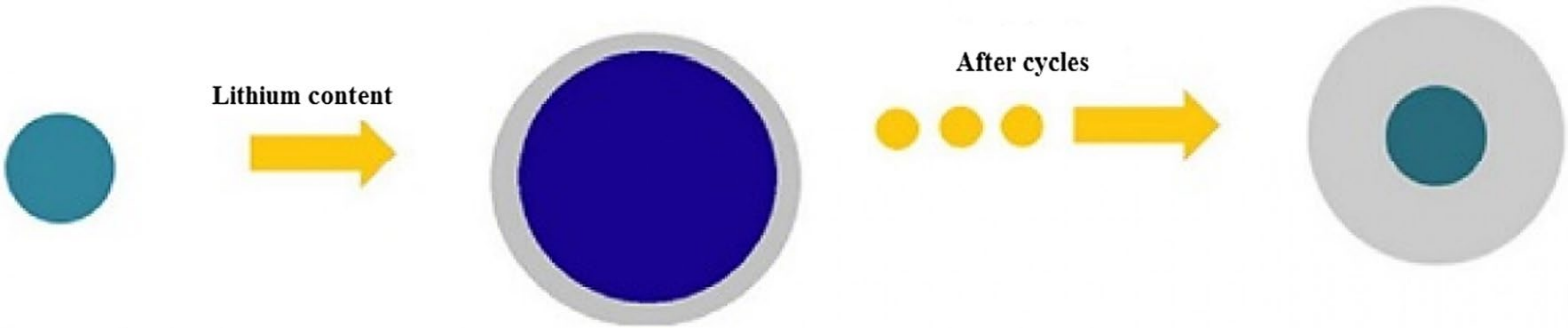
How the SEI layer grows [19]

**Fig. 13:**
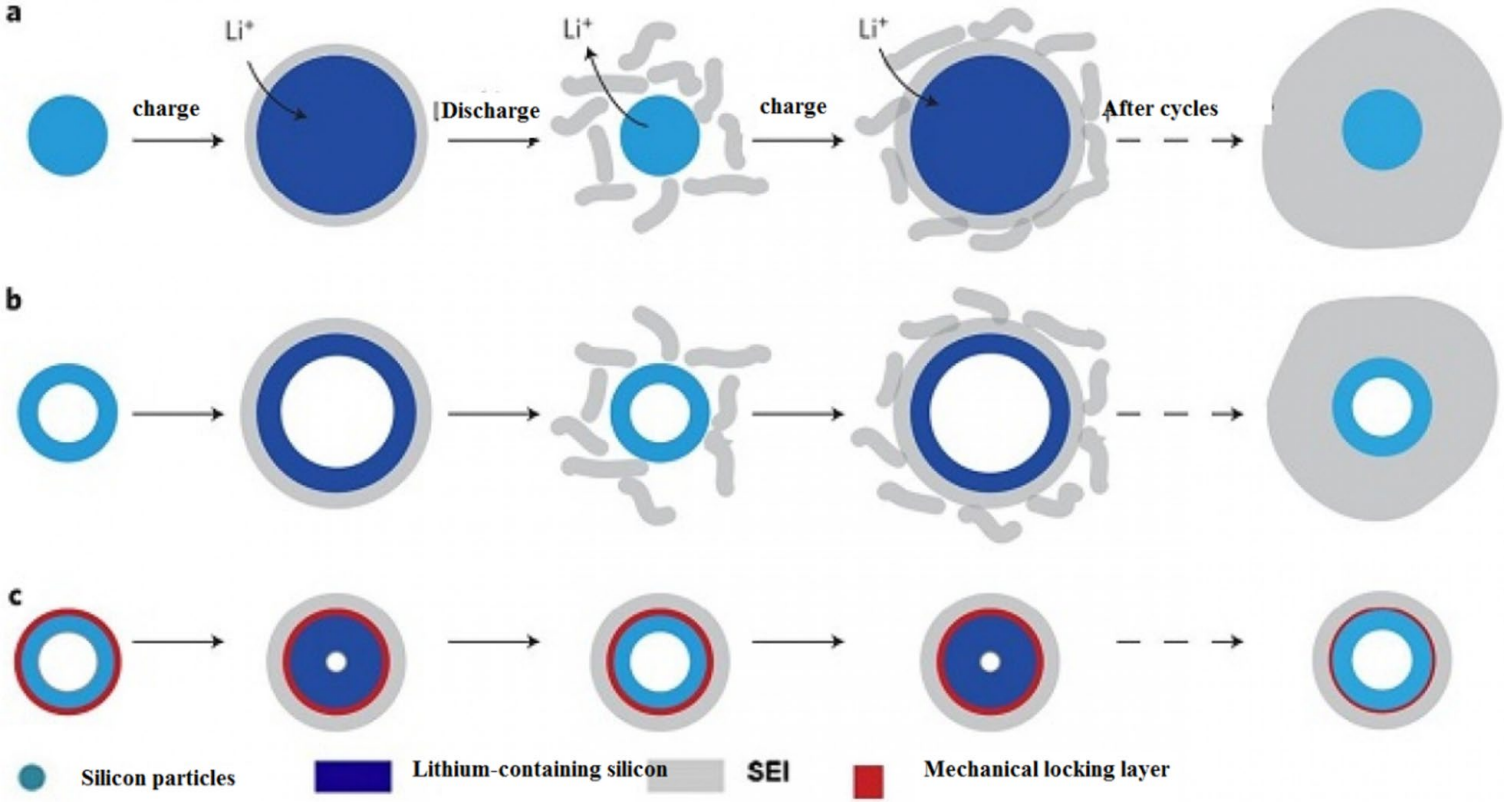
Shows the growth of SEI in different conditions [20]

In general, for anodes less than one volt relative to lithium metal, the electrolyte is unstable and SEI is formed. Hence, SEI is formed in the silicon anode, which has a potential of 0.3 to 0.4 higher than lithium [112–115]. Unfortunately, because silicon changes volume and breaks down, new levels of silicon are exposed to the electrolyte, so the electron reaches the electrolyte and a new SEI is formed on these new surfaces. As a result, capacity is constantly reduced during work cycles. It is necessary to say this, because tests are often performed on lithium metal, voltages relative to lithium are measured in all battery articles [69]. In silicon nanomaterials, because of the higher chemical activity, it is even more susceptible to the formation of SEI. In the case of nanomaterials, it is true that they do not break down, but they do change volume. According to Fig. 12, this volume change causes SEI to grow continuously and we see the disadvantages of SEI growth, such as reduced capacity and power, and so on. Figure 13 of section a better illustrates the reason for SEI growth in nanomaterials. If we have the cross section of a nanowire (or nanoparticles, etc.) in the initial state without lithium, shown on the left side of the figure, during charging, because the silicon is lithium-containing, its volume increases and due to electrolyte instability at the same time an SEI layer is formed on the nanowire [116–119]. Now during discharge, the lithium comes out and the particle shrinks while the SEI does not shrink. This causes the SEI to crumble under stress (or even in the second stage of silicon enlargement under lithium ionization, at which point stress occurs and the SEI crumbles because the exact boundary between the SEI and the particle does not exactly match). Therefore, when recharging (lithium ionization), a new SEI layer is formed again. Repetition of this cycle leads to continuous SEI growth and we have problems with its growth, while in graphite SEI would not grow without a slight change in its volume. It should be noted that what has been said about SEI and silicon also applies to other alloy anodes [70, 120–123]. As seen in section b, this problem also exists for silicon nanotubes, but if we can somehow prevent the silicon from coming into contact with the electrolyte from the beginning and changing its volume in the vicinity of the electrolyte, this problem will be solved.

**Fig. 14:**
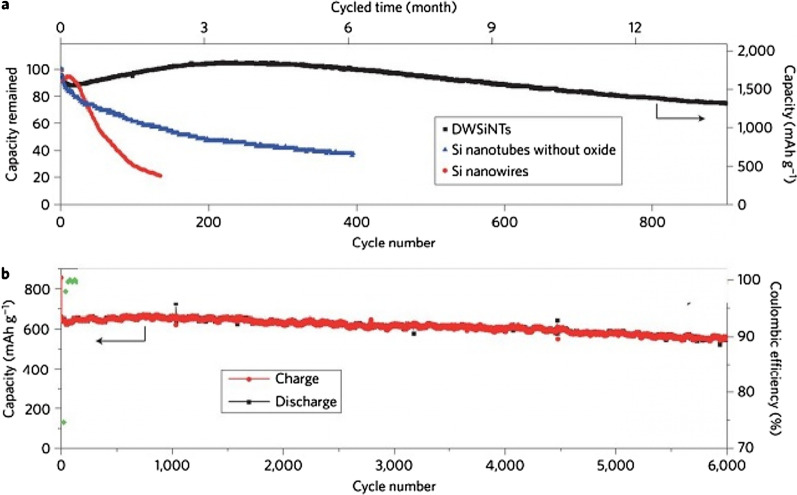
**a** Comparison between the cycle life of black, blue and red for oxide-coated, **b** oxide-free and non-oxide nanotubes, respectively [21]

**Fig. 15:**
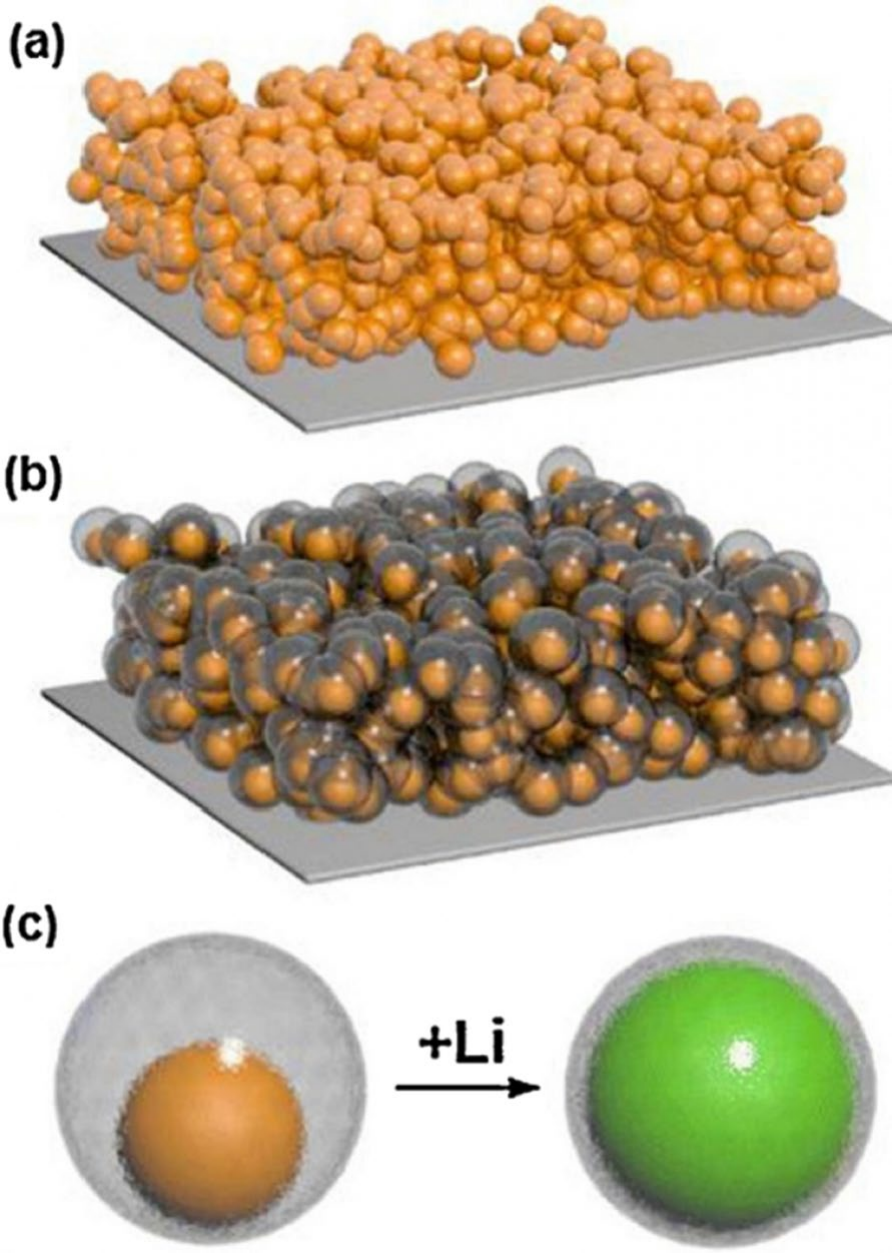
**a** Display of electrodes made of silicon nanoparticles. **b** Electrode display made of silicon nanoparticles with carbon coating and hollow structure. **c** The structure of the core-hollow shell used in **b**, the silicon is inside the hollow carbon and its volume change is observed during lithium ionization [10]

Wu et al. [18] used a mechanical locking layer as shown in Fig. 13c; this layer, which is made of silicon oxide, prevents the change in the outer volume of the nanotubes due to its mechanical strength. Thus a stable SEI is formed without changing the volume (a stable SEI layer like graphite). This oxide layer is the conductor of lithium ions so it does not cause a problem to react. The necessary space for volume change is also provided through the inner wall of the nanotube. So there is no problem of crushing. Because the study showed that the electrolyte does not penetrate into the nanotube, there is no contact between the electrolyte and the inner wall of the nanotube. All these advantages make it offer a long cycle life and good power. Figure 14 (shown in this figure with DWSiNT) shows part of the deep discharge cycles of this sample. In deep discharge, the cycle life is always reduced faster. However, it is observed that after 900 cycles, the prepared sample still has a good capacity, but the normal nanotube and nanowire samples lose their capacity rapidly. Discharged) For the plotted sample, it shows that the capacity maintains its capacity even after this relatively high C rate even at up to 6000 open cycles. In another example [19], a core–shell structure is prepared, as shown in Fig. 15c, a carbon coating is used with silicon nanoparticles inside the carbon coating. The thickness of the carbon coating is in the range of 10 nm and includes 100 nm silicon particles. The carbon shell is provided with enough space to easily change the volume of the silicon nanoparticle, as shown in Figure c. On the other hand, silicon nanoparticles are attached to the carbon shell from one point, so electron and ionic transitions take place in it, because carbon is in the vicinity of the electrolyte and not silicon, like graphite, a stable SEI is formed without crushing because the change in volume of silicon is not transferred to carbon and from carbon to SEI, so similar to the figure in Fig. 12, it has a long cycle life. If we use silicon nanoparticles normally, in addition to the SEI problem, as we saw in the previous article and Figure a, it shows that there is no empty space between the silicon nanoparticles to change the volume, so there is stress between the particles when they change volume, but when Using this hollow structure (Figure b) there is no longer any stress between the particles.

This anode has other advantages in addition to the SEI problem, compared to the sample in Fig. 12. One advantage of nanoparticle synthesis advantages over nanotubes, and more importantly, the use of nanoparticles compared to nanowires, is well compatible with the slurry method, which is the conventional method of preparing electrodes in batteries.

## Introducing LTO Anode

**Fig. 16:**
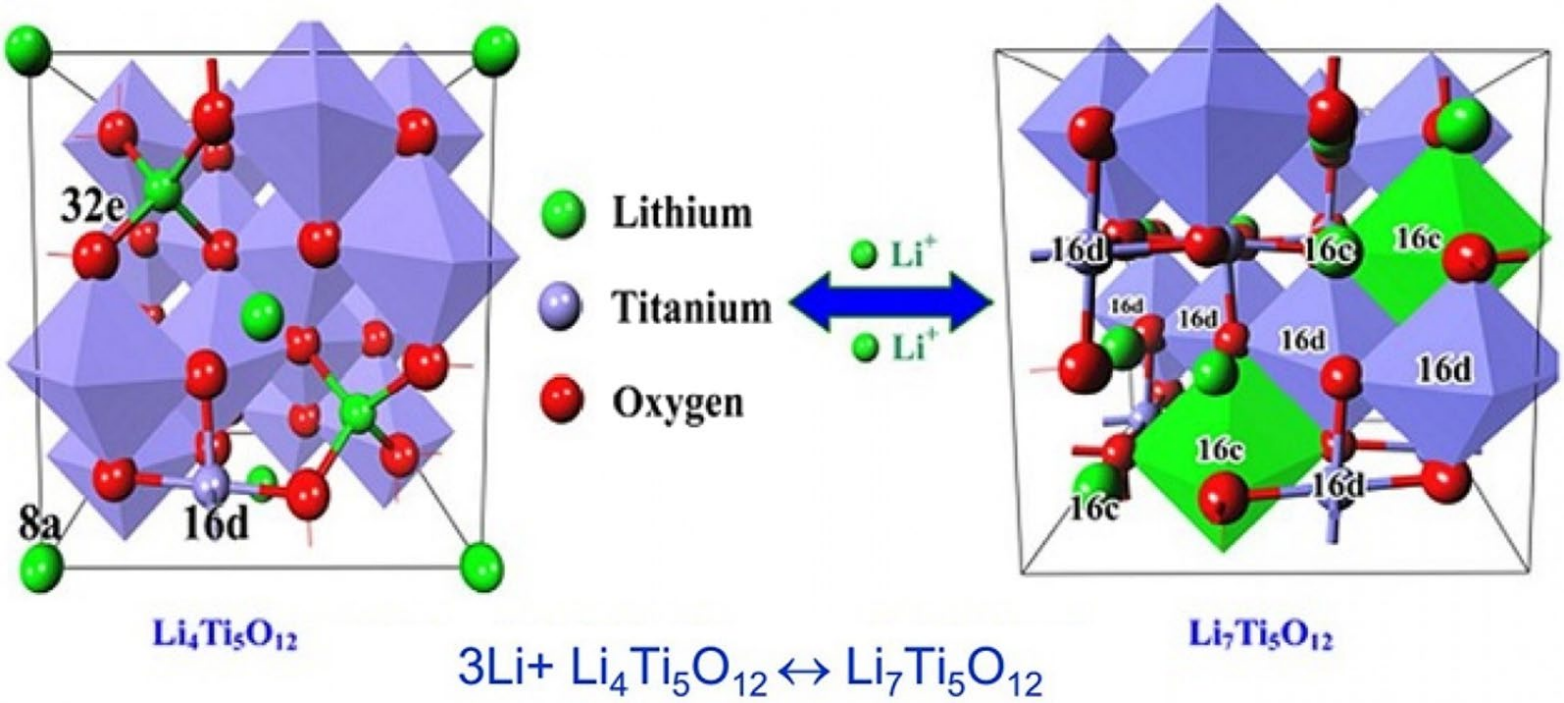
Shows the structure and entry of lithium ion in LTO along with its reaction [74]

**Fig. 17:**
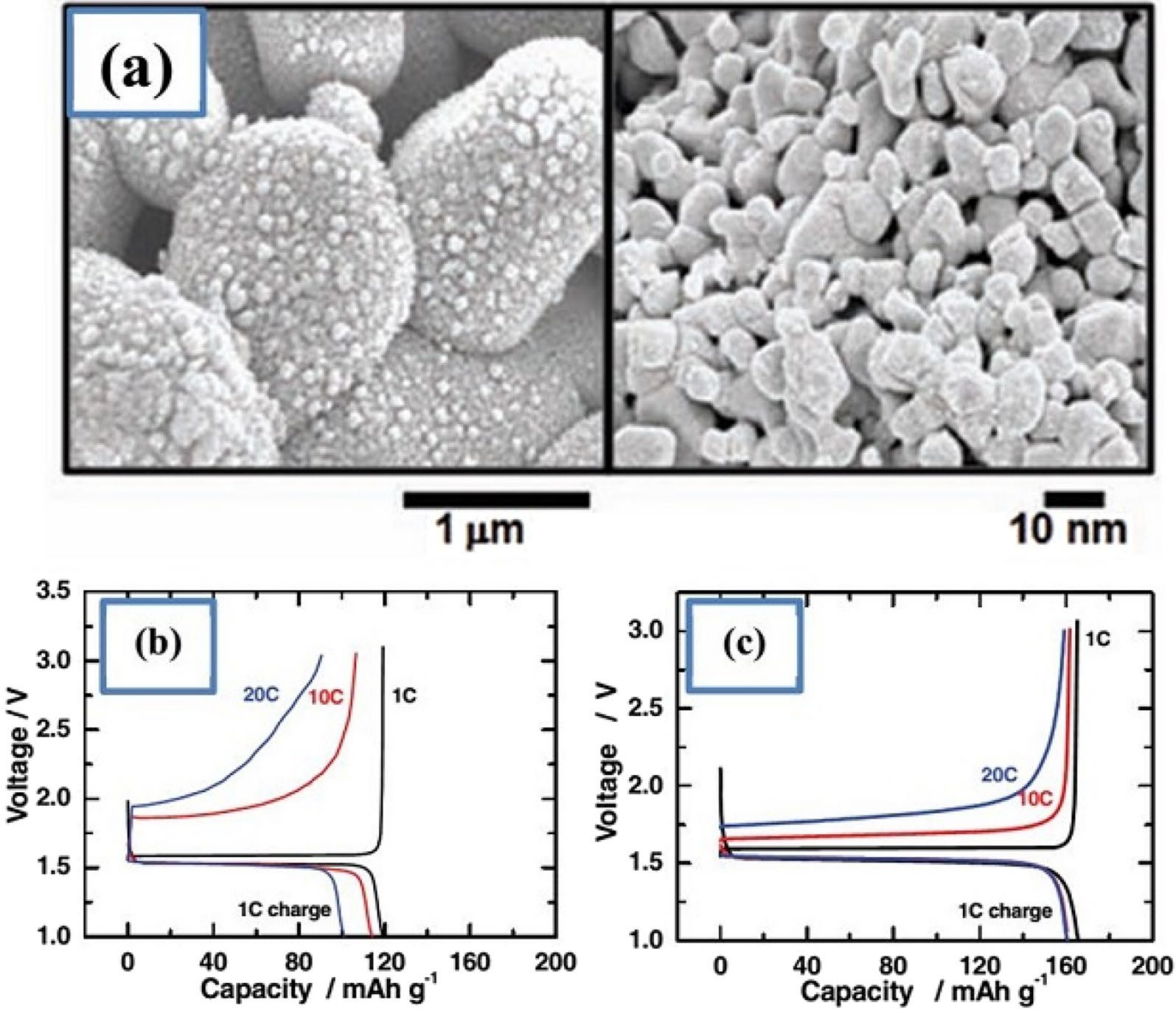
**a** Displays the nanostructure discussed including Nano primary nanoparticles, **b** charge–discharge curve for ordinary micro particles, and **c** for a-shaped particles [74]

So far, we have talked about two types of graphite anodes and alloy (silicon) anodes. Another anode that is very popular is the anode with Li4Ti5O12 compound, which is called LTO for short. This anode is like intercalation graphite [29, 124–127]. Figure 16 shows the structure and reaction of this type of anode. The LTO anode has a limited capacity of 175 mAh/g (compared to 300 graphite and 4000 silicon). The voltage of this anode is also about 1.5 V compared to lithium metal according to Fig. 17 (the lower the anode voltage, the higher the battery voltage). This high voltage and low capacity both make this anode have very low energy, but it is still one step ahead of silicon in commercial terms. One of the most important features of this anode is the safety issue, because in electric vehicles there are unpredictable conditions, and the other is the long cycle life, and finally its power [72, 73, 128–130].

Due to the fact that the voltage of this anode is high, it is in the range of electrolyte stability according to Fig. 17, so SEI is not formed. On the other hand, as shown in Fig. 17, there is enough space for lithium ions in this composition and it does not change volume, while even in graphite, some volume change is seen due to the entry and exit of lithium. Unlike the previous two anodes, lithium ions (not lithium atoms) are stored in this anode, and the oxidation reaction is due to the conversion of titanium to 3-valent titanium, not to a change in lithium capacity [28, 74, 75, 131].

This battery, because it has neither SEI nor volume change, maintains the capacity well and has a very long cycle life (more than graphite) of about 20,000 cycles. Because it is an oxide compound and is very safe due to the lack of volume change. Because it does not have SEI, its power is not bad either, only its lithium ion diffusion coefficient is low and its electron conductivity is poor. To solve this problem, they provide LTO nanostructures. Because this anode did not have SEI from the beginning, when it becomes Nano, it does not have the problem of forming more SEI, so it does not have more nanomaterial activity [32, 35–37].

It has been observed that nanoparticles cause the LTO anode to charge and discharge within 5 min (12C). To prepare the nanostructure, first titanium oxide nanostructure is prepared and then reacted with a lithium source material when heated. This is also an advantage of LTO, as the preparation of TiO_2_ nanostructures is very popular. Due to the problem of low volumetric density and agglomeration of nanomaterials, micron secondary particles made from nanoscale primary particles are more useful [33, 38, 58, 59].

Figure 17 shows part an of this nanostructure. As can be seen, from the controlled community of smaller particles measuring 10 nm, larger micron particles are formed. According to the comparison of parts b and c in Fig. 17, it is quite clear that this nanostructure is superior to ordinary micron particles, because it has less capacity and potential (especially in discharge). From this nanostructured anode, a battery is made and it is observed that this battery is superior to the battery with graphite anode both in terms of cycle life and power, which is not given due to the brevity of these curves [75]. The benefits of Nano-LTO have been well documented in many articles, but what makes it stand out is an important discussion of proper engineering of the structure, proper synthesis method, and how to use the conductive material to improve conductivity for further improvement. The future will be talked about. In addition, it is not disputed that nanotechnology is useful for LTO, but many of the phenomena that occur at the nanoscale for LTO are discussed so that some are not fully understood.

**Fig. 18:**
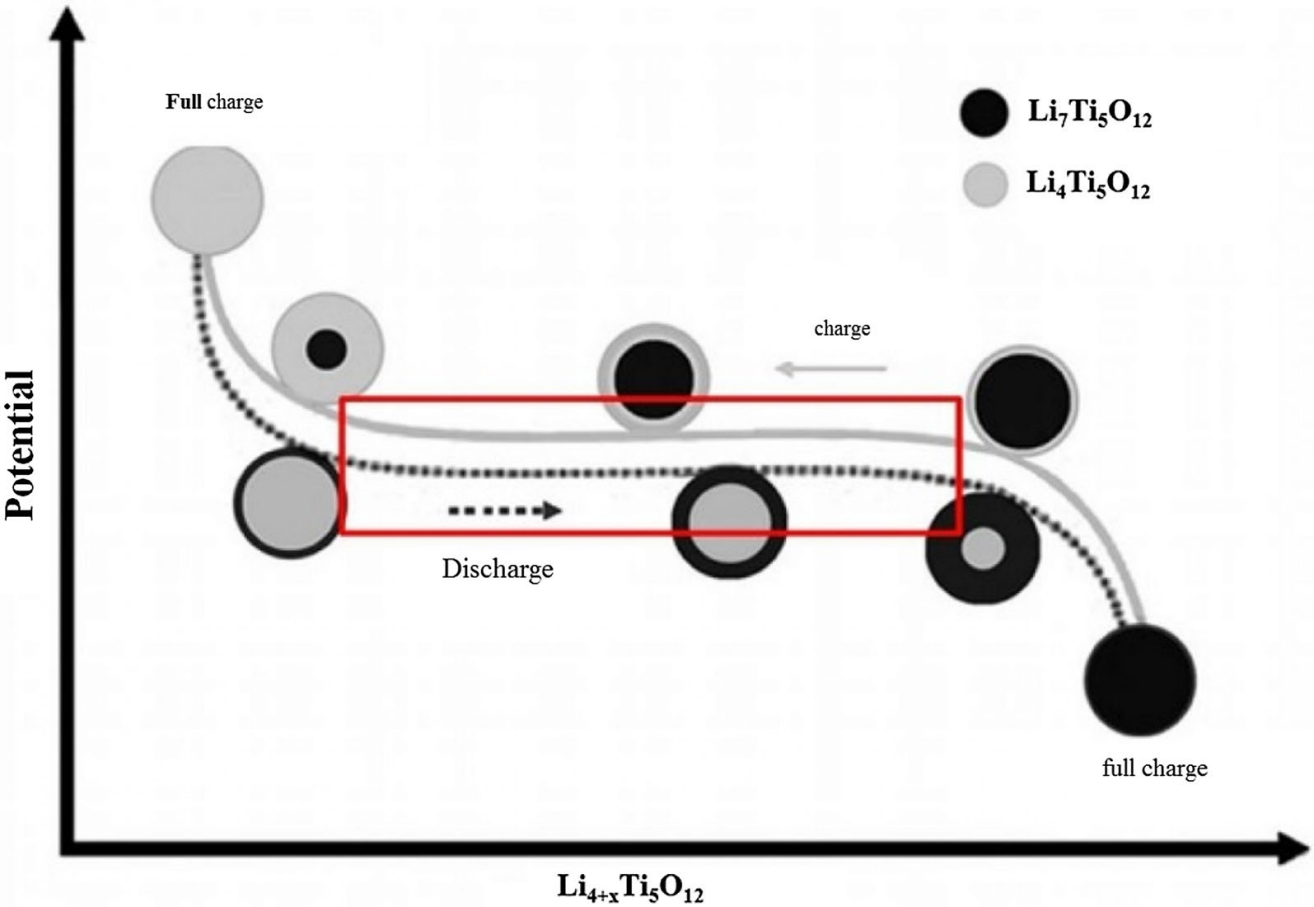
Shows the linear curve range at the LTO anode in the charge–discharge axis [75]

Another phenomenon that occurs at the nanoscale is the change in charge–discharge curves for the LTO anode. This anode provides a constant voltage over a wide range of capacities (red box in Fig. 18). When LTO ions are Nano, the constant voltage range decreases until after a critical limit (in the range of a few nanometers) there is no longer a constant voltage range [76].

One of the things that happens on the surface is the insertion of more lithium ions into the surface layers. In the surface after insertion, we reach the formula Li_8.5_Ti_5_O_12_, which is 1.5 mol more than the inner layers with the formula Li_7_Ti_5_O_1_, but in the micron material, because the percentage of surface is not high, it shows its effect, but for Nano, because the amount of surface is large, the effects are large. There are several on the charge–discharge curve.

## TiO_2_ Anode

There is also a TiO_2_ anode from the LTO family. These anodes are easier to synthesize, and because they do not want to react with heat-induced lithium ion precursors, they do not have heat-induced problems such as nanomaterial growth. In addition, according to the chemical formula, titanium oxide has a capacity of twice the amount of 335 mAh/g (LTO). The general response of these anodes is ${\text{TiO}}_{2} + x{\text{Li}}^{ + } + xe^{ - } \leftrightarrow {\text{Li}}_{x} {\text{TiO}}_{2} .$

TiO_2_ has four types of phases or crystallographic structures (different atomic arrangements) known as Brocket, Anastasi, Rutile, and (TiO_2_ (B). The Brocket phase does not matter to the battery. Antara and rutile, which are very popular phases, are important as anodes. Phase (TiO_2_ (B) performs better than others due to its atomic open space and suitable channel for ion transport, and is the most important [75, 76, 132–136].

If we consider the theoretical capacity based on the chemical formula (one mole of lithium ion per mole of TiO_2_), it is equal to the above value, but based on the phase and position that can be placed according to the lithium ion crystal lattice, different theoretical capacities for different phases have been reported; for example, for anisate, according to network sites, the half-capacity is high, 0.5 mol of lithium ion per mole of TiO_2_, 167 mAh/g.

**Fig. 19:**
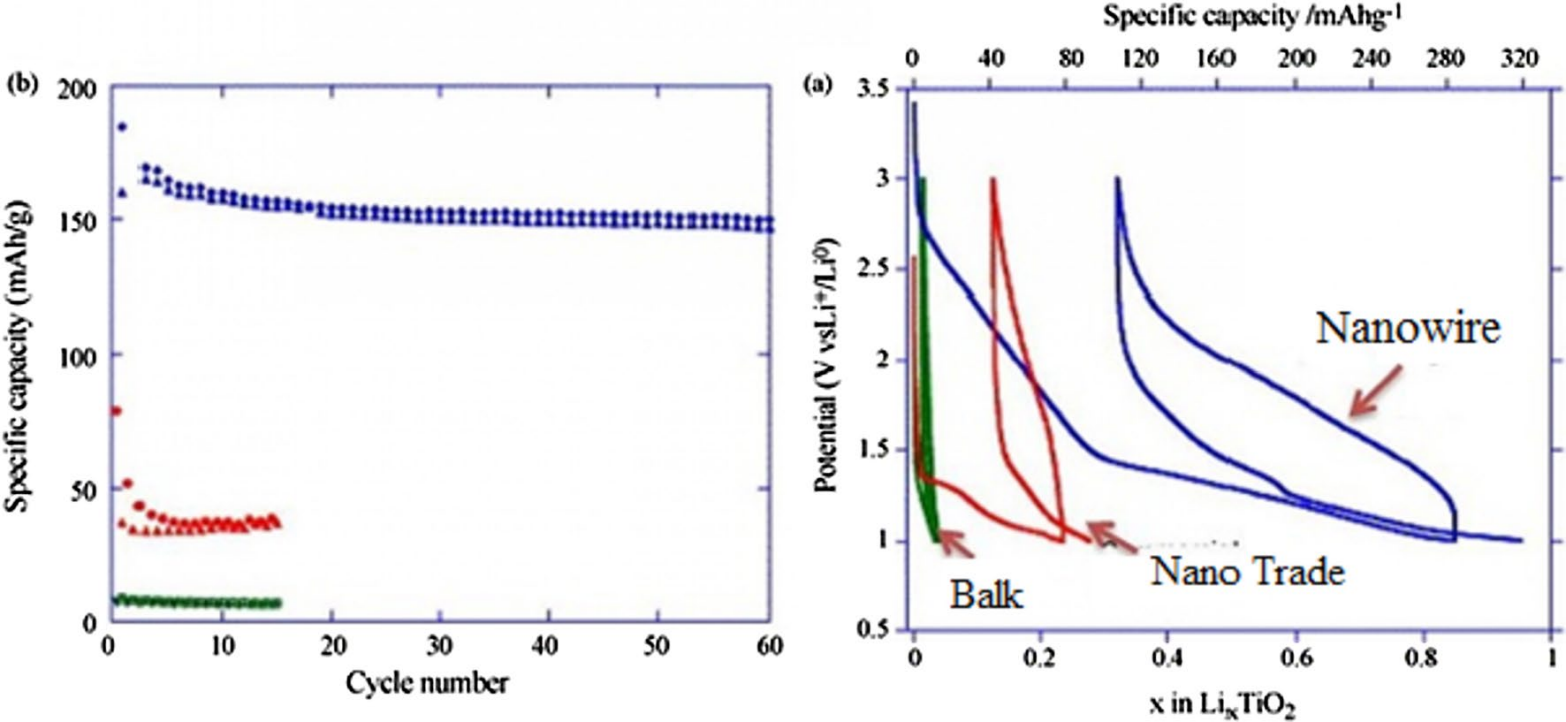
**a** Charge–discharge curve for the first time, **b** cycle life [77].

**Fig. 20:**
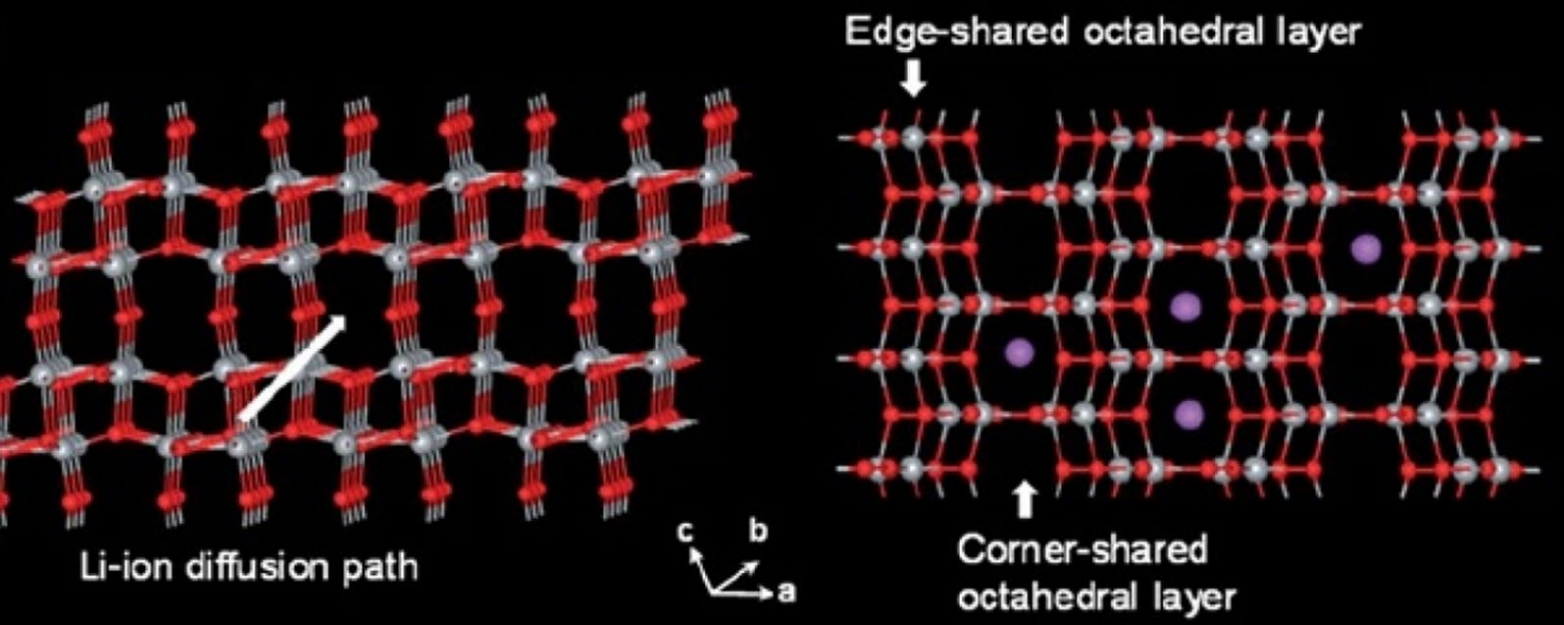
Showing the atomic structure of the phase (TiO_2_ (B) [77]

Because all of these phases have poor ionic conductivity, the nanoscale is very effective in increasing both power and capacity. What is interesting is that the nanostructured capacity of Anastasi is more than the theoretical capacity based on the position of the network, but it is definitely less than the theoretical capacity of Formula 334 in all phases. Rutile in micron mode can only store 0.1 mol of lithium ion per grid unit. In rutile, lithium locations are located throughout the network, but the diffusion coefficient in the direction of the *c*-axis is one order of magnitude greater than that of the ab plate [137–141]. The lithium atom penetrates well in the direction of the *c*-axis, but must be diffused throughout the space by penetrating the ab plane, and because the diffusion velocity is low in the ab plane, lithium ions accumulate in the c channel, causing a charge repulsive force. Lithium ion positive is generated. This repulsive force prevents more ions from entering the network. As an interesting result of the Nano effect, it has been shown that when the dimensions of rutile become Nano, the capacity reaches 0.8 mol of lithium ion, which has a reversible capacity during different cycles, which reduces the penetration distance and the effect of its quadratic power [142–146]. There is no repulsive force. Figure 19 shows the charge and discharge curves and the cycle life for rutile bulk (micron), commercial rutile micro particles, and rutile nanowires. As can be seen, nanowires show good cyclic longevity and capacity. The shape also confirms that the shape of the nanomaterials also affects the performance of the anode. Morphology such as nanoparticles, nanowires, etc. differ in both capacity and life cycle and power, but the type of morphology alone is not decisive but the geometry of the structure that determines the performance (in the future about the geometry of the structure for all active materials for example, nanowires connected to a current collector, nanowires mixed with graphene, and insulated nanowires each present different results. In addition, there are test conditions and C rate and many other factors [77]. Phase (TiO_2_ (B), which is newer than other phases, offers the best power and capacity due to its suitable channels for lithium ion transport [147–151]. Figure 20 shows the structure of the penetration site. The capacitance can be significantly increased. This phase offers the best power and capacity among all titanium anodes including LTO, so that by Nano partying it in just 4.5 s, the anode can be charged or discharged with a capacity of 73% of theory. We do not have volume change in this anode either.

## Quasi-capacitive Capacity

**Fig. 21:**
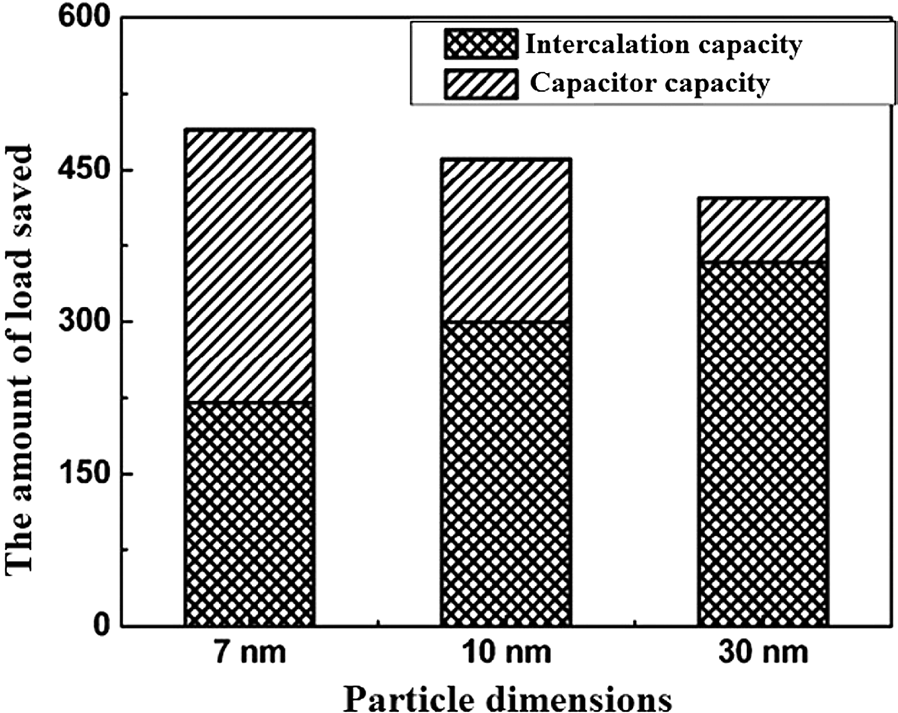
Demonstration of input and super capacitor capacities in titanium oxide [78]

So far, it has been discussed about the storage capacity of lithium ions in the form of a degree in the nuclear network, and this capacity is improved in the nanoscale due to the reduction of the penetration distance, and so on [152–155]. But one of the interesting phenomena that occurs for these anodes at the nanoscale is the storage of lithium ions at the surface due to the large surface-to-volume ratio. This type of storage is different from the insert and alloy capacity mentioned so far. This type of storage is very fast because it does not require penetration, and also because it does not create stress and the like, it has the best cycle life and power compared to other lithium storage methods [156–158]. Of course, this type of capacity generates less energy. This capacity is discussed in more detail in the topic of super capacitors. The Fig. 21 shows a comparison between the storage capacity of LTO capacity in three different Nano dimensions [78]. According to Fig. 21 in small Nano dimensions, this capacity is significant and decreases significantly with increasing dimensions. It should be noted that capacitive capacitance is not only related to titanium oxide compounds but is also present in many other active substances that are mentioned when introducing them.

## Introduction of Exchange Anodes

**Fig. 22:**
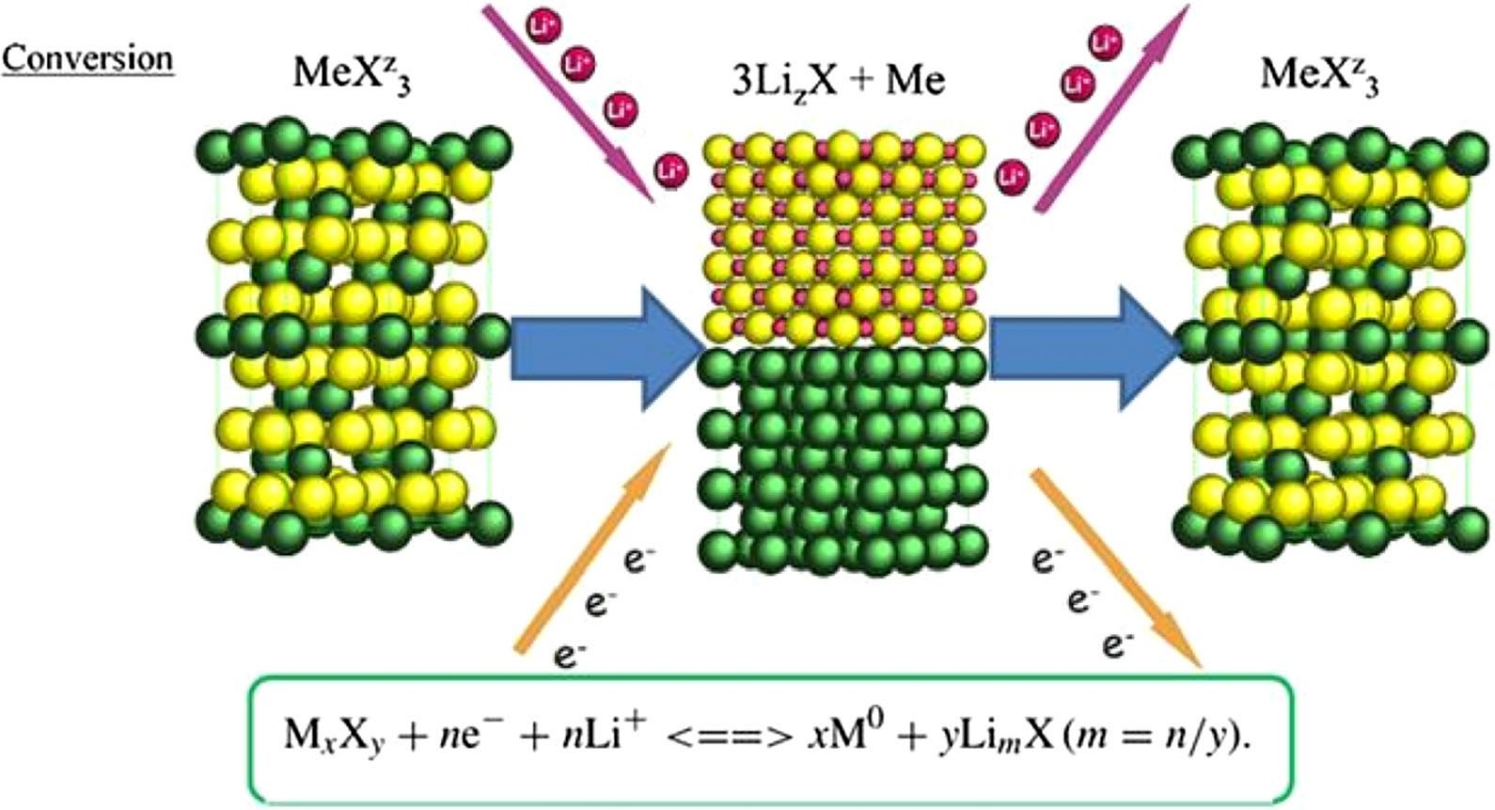
Shows the structure and entry of lithium ion with its reaction [22]

**Fig. 23:**
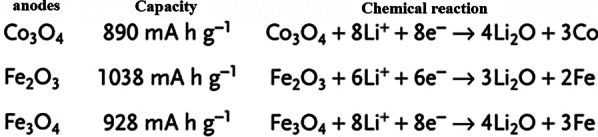
Shows the reaction and capacity of a number of conversion oxide anodes [30, 55]

So far, we have talked about two types of insert electrodes and alloys. The third type of electrode operation is based on a conversion reaction. Figure 22 shows the mechanism and reaction of this type of electrode. In this form, M (or Me) is an intermediate element that is oxidized, and X is an anion such as oxygen, sulfur, and the like [159–161]. The advantage of these anodes is that for every MxXy unit, *n* lithium ions (*n* more than one) are involved in the reaction, whereas in the graphite insert anodes we see one lithium ion for every 6 carbon atoms stored in titanium compounds. A maximum of one lithium ion is stored per TiO_2_ formula unit. But in the exchange anode, for example, for CoO and FeO, the value of n is equal to 2, and in Co_3_O_4_, the value of n is equal to 8. Figure 23 shows a number of exchangeable oxide anodes with their reaction and capacity [22, 30, 55].

## Exchange Anode Problems

**Fig. 24:**
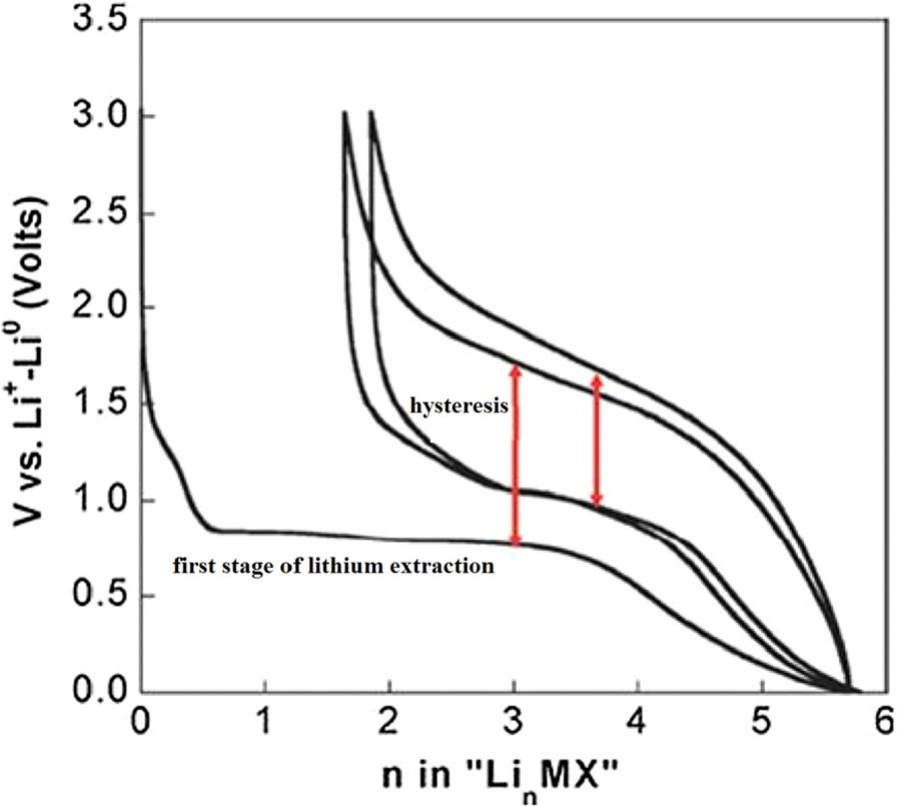
Show charge–discharge curves of exchange anodes [79]

Exchange anodes are very similar to alloy anodes, as alloys have problems with volume change, fragmentation, and SEI formation. In these anodes the ionic and electron conduction is low, and in addition the exchange rate is slow. This low speed leads to high potential during charging and discharging. At these potentials, there is a large difference between the charging and discharging voltages, called hysteresis, which is shown in Fig. 24 with a red arrow. This figure shows that in the first stage of lithium extraction, the anode behavior is significantly different from the next stage of charge and discharge. The hysteresis in this type of anode is up to one volt, while in the graphite and LTO anode it is about 0.2 V. This hysteresis is mostly due to activation polarization [78, 79].

## Nano Sizing Effects

**Fig. 25:**
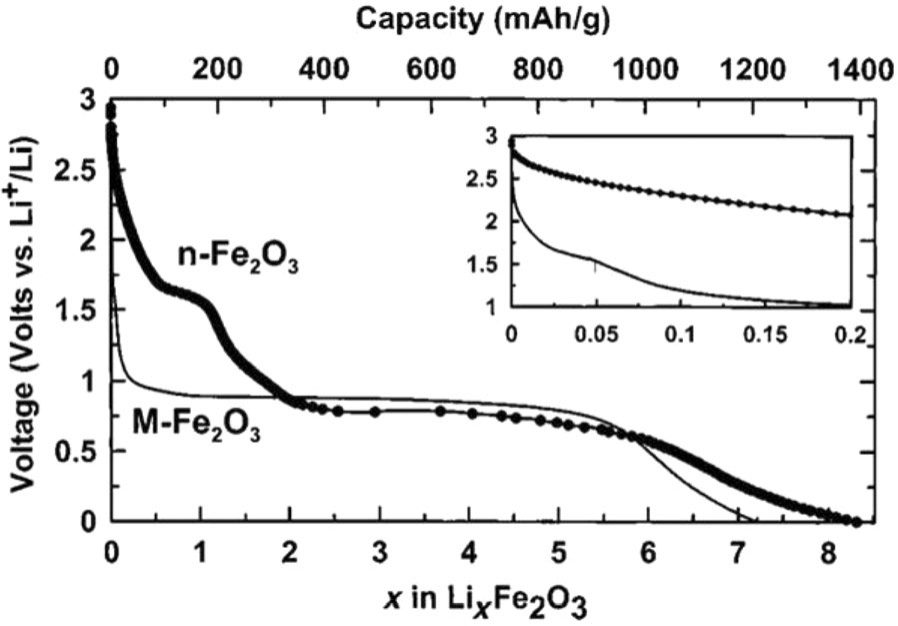
Demonstration of lithium ionization for *n*-Fe_2_O_3_ and micro-M-Fe_2_O_3_ [80]

**Fig. 26:**
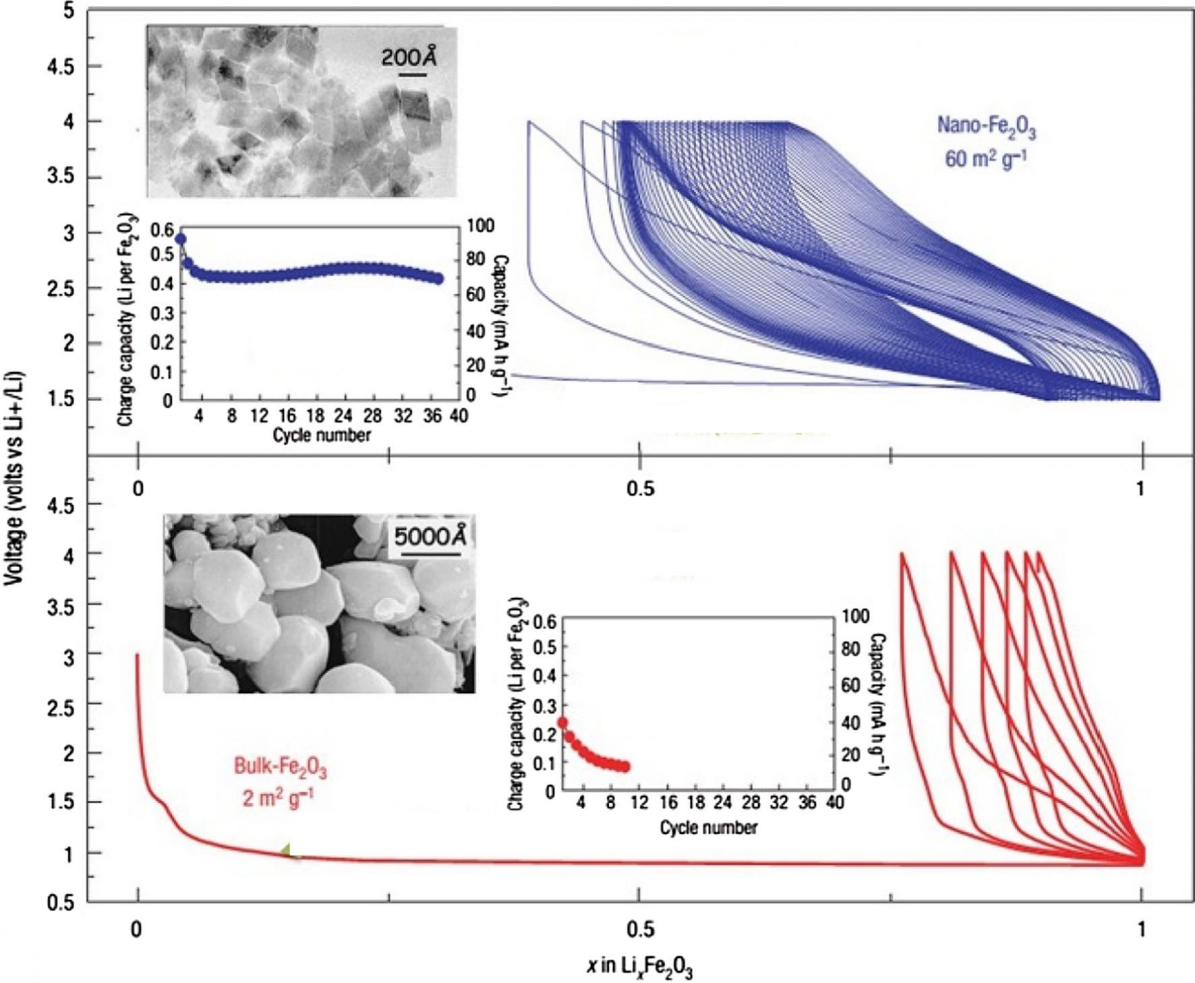
Display of SEM images, charge–discharge curves and cycle life for Nano-iron oxide and bulk [80]

Figure 25 shows the lithium ionization behavior (in the test mode, against lithium metal) for anodes made of fine nanoparticles (20 nm) and micro-nanoparticles (500 nm) of iron oxide (SEM) images of these particles in Fig. 26. Available it can be seen that the capacity of the Nano anode is slightly higher. More importantly, it can be seen that the charge–discharge behavior of these two anodes is very different from each other, which is examined in Fig. 26. Figure 26 shows the charge–discharge curves in different cycles as well as the cycle life for the same samples in Fig. 25 to determine the reason for the difference in charge–discharge curves in Fig. 25. Note that instead of capacity, lithium that enters and leaves (which, according to the arguments, represents capacity) is used. In the charge–discharge curves of Fig. 26, lithium ionization continued only up to 1 mol because its purpose was to investigate the behavior in this range of lithium ions. As can be seen, the effective surface mass for the material is only 2 m^2^/g while for the Nano it has an effective surface area of 60 m^2^/g, which indicates how much higher the effective surface area is at the Nano. The difference between Nano and Nano performance is also quite clear. As shown in Fig. 26, the amount of reversible lithium (which can be removed during charging) for Nano is much higher than the corresponding amount for bulk. This shows that the capacity that can be recovered after the initial charge is much better in Nano than in micro. Also, according to the same figure, in the next consecutive charge-discharges, the amount of lithium entering and leaving is less than 0.25 (from 0.75 to 1), while for Nano, the amount of lithium entering and leaving is more than 0.5 (the amount of lithium ion). In the composition it has changed from the range of less than 0.5 ions to 1 ion), according to this, the capacity offered in Nano is much more than bulk. In the micron-sized anode of Fe_2_O_3_ (hematite), before the exchange reaction begins, about 0.1 mol of lithium ion per mole of oxide compound can be stored in the lattice, but above this critical limit, the exchange reaction takes place; on the other hand, when we increase the dimensions of iron oxide particles to 20 nm, the amount of lithium stored in degrees reaches 1 mol, which causes a volume change of only about 1%. Of course, about 0.5 mol is reversible (Fig. 25). In fact, the type of storage mechanism (input, exchange, etc.) changes and the type of mechanism affects the shape of the charge–discharge curve. The above paragraph indicates that when the oxide dimensions enter the Nano field, the storage mechanism is also affected. So far it has been said that Nano makes volume change easier without failure, but here it can be seen that even Nano has reduced the amount of volume change from a few percent for the exchange reaction to one percent for a degree reaction. The reason for this change is the storage mechanism for iron oxide due to thermodynamic problems. The opposite happens for the Co_3_O_4_ anode because it is kinetic and is related to the current density (the current density is obtained by dividing the current by the surface); when the current is constant, in the Nano-dimensions, because the surface is higher, the current density decreases and the Co_3_O_4_ anode shows exchange behavior, but in the micro, due to the high current density, the anode shows the insertion behavior [76–79].

It can be seen from Fig. 26 that at the nanoscale, the cycle life is also much better than bulk. The reason for the improvement of these expressed properties is the ease of volume change and release of stress, ionic and electronic transitions are easier due to the reduction of the penetration distance, which was expressed in this series of articles. Due to high hysteresis, less attention has been paid to compounds with hysteresis [22, 51–54, 80].

## Nanomaterials in Batteries

Nanomaterials have been widely applied in the life sciences, information technology, the environment, and other related fields. Recently, nanostructured materials have also attracted attention for application in energy storage devices, especially for those with high charge/discharge current rates such as lithium ion batteries. The development of next-generation energy storage devices with high power and high energy density is key to the success of electric and hybrid electric vehicles (EVs and HEVs, respectively), which are expected to at least partially replace conventional vehicles and help solve the problems of air pollution and climate change. These energy storage technologies will rely on innovative materials science, i.e. developing electrode materials capable of being charged and discharged at high current rates. Generally, the potential advantages of nanostructured active electrode materials can be summarized as follows: new reactions can be used that are not possible with bulk materials; a larger electrode/electrolyte contact area, leading to higher charge/discharge rates; short path lengths for both electronic and Li ion transport (permitting operation even with low electronic or low Li ion conductivity, or at higher power); etc. Here, we review some recent experimental results that show the advantages of nanostructured active electrode materials [147]. Table 2 summarizes the nanotechnologies that are used to produce nanomaterials, such as mechanical ball milling, chemical vapour deposition, the template method, electrochemical deposition, hydrothermal reaction, dehydration, sintering, pulsed laser deposition, ultrasound, sol–gel synthesis, and micro emulsion.

**Table 2:** Techniques and nanomaterials used in batteries

Techniques	Nanomaterials	Lithium storage capacity for electrode materials	References
Mechanical milling MWNT made by chemical vapor deposition	SWNT	600 mAh/g	[162]
Mechanical milling MWNT made by chemical vapor deposition	Sn	670 mAh/g (1st cycle)	[163]
Mechanical milling MWNT made by chemical vapor deposition	MWNT-Sn	570 mAh/g (1st cycle)	[163]
Mechanical milling MWNT made by chemical vapor deposition	MWNT-SnNi	512 mAh/g (1st cycle)	[163]
Mechanical milling	Ag_3.64_Fe_15.6_Sn_48_	530 mAh/g (1st cycle)	[164]
Mechanical milling	Ag_3.64_Fe_15.6_Sn_48_	420 mAh/g (300 cycle)	[164]
Chemical vapor deposition	MWNT	340 mAh/g	[165]
Electrochemical deposition	Cu_6_Sn_5_	400 mAh/g (30 cycle)	[166]
Mechanical milling	Si (78 nm) composites	1700 mAh/g	[167]
Sol–gel based template synthesis	V_2_O_5_ (nanowires)	147 mAh/g	[168]
Sol–gel synthesis	LiM_*x*_Fe_1−*x*_PO_4_ (M = Mg, Ti, Zr) (40–150 nm)	160–165 mAh/g at C/8	[169]
Hydrothermal reaction	TiO_2_ nanotubes	170 mAh/g (1st cycle)	[170]
Sintering	WS_2_ nanotubes	915 mAh/g (1st cycle)	[171]

**Fig. 27:**
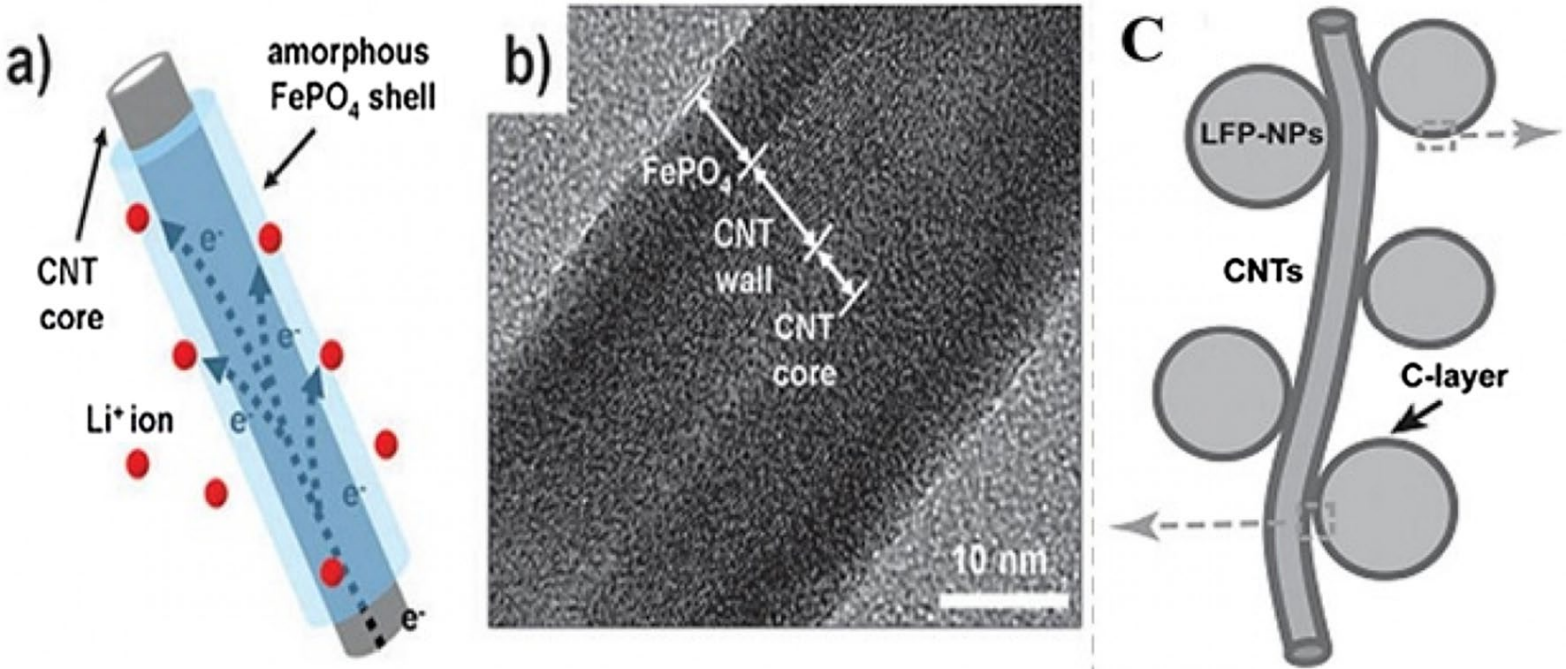
**a**, **b** With carbon nanotube core and LiFePO_4_ wall, and Figure **c** LiFePO_4_ nanoparticles attached to carbon nanotube [3, 93]

The first group of applications of nanotechnology in batteries is itself divided into two categories: the first group nanoscale the active substance in the electrode, the second group use nanotechnology to improve the performance of electrodes (cathode or anode) by adding nanomaterials other than the active substance, or the use of Nano coatings. For example, Nano-dimensional additives such as Nano carbons, graphene, carbon nanotubes, etc. have better electron conduction, or the use of Nano-thick coatings on the active material to prevent unwanted reactions with the electrolyte, stress modulation, provide stability and …. for it. For example, for a LiFePO_4_ cathode, the amount of electron conductivity is poor. Conductivity is improved by using a conductive carbon coating on its particles or by using a conductive carbon material as an additive, A Nano-thick coating of oxide is used [83, 94, 172–177]. For example, for a LiFePO_4_ cathode, the amount of electron conduction is poor, Conductivity is improved by using a conductive carbon coating on its particles or by using a conductive carbon material as an additive, or the LiCoO_2_ cathode is unstable at high currents in the vicinity of the electrolyte, using a Nano-thick oxide coating to stabilize it [162, 163, 178, 179]. If we want to illustrate the field of nanotechnology in this category with an example, in the same LiFePO_4_ cathode it has been shown that carbon coating increases conductivity and consequently power, capacity, etc., but one of the areas of research is how to create this coating. Be cheap, effective, etc.; therefore, research in the field of synthesis methods is very important. On the other hand, how to add the same coating and additives to be more effective, so the engineering and architecture of nanostructures is one of the important areas of research and the preparation of these engineered structures is also an interesting issue. Consider Fig. 27 to clarify the matter. This figure shows two types of Nano-engineered structures for the LiFePO_4_ cathode that use carbon nanotubes to improve conductivity. In addition to differences in performance, each of these structures has a different synthesis method, which indicates the importance of synthesis.

Silicon has attracted tremendous attentions as one of the most promising candidates for the next-generation Li-ion batteries (LIBs). Compared to the traditional graphite anode, it has many obvious advantages such as large capacity, high abundance and environmental friendliness [1–4]. Unfortunately, due to the huge volume expansion (~ 300%) in lithiation, silicon particles are pulverized and solid electrolyte interphase (SEI) layers formed on their surface are unstable. Therefore, the long-term cycling stability of silicon anode is poor [5–8]. Moreover, the low intrinsic conductivity of Si causes unsatisfying rate-capability [9–12]. Thus, a large amount of Si/metal (e.g., Ag, Cu, Al, Sn) composites have been developed to solve the low conductivity [13–16]. However, the large volume expansion cannot be relieved effectively. On the other hand, carbon Nano layers are coated on the electrode materials to increase their conductivity, enhance their mechanical strength and provide them stable interfaces with electrolyte. Therefore, various conformal carbon layer coated silicon (Si@C) nanostructures are developed. For example, Si@C with core–shell structure are formed by pyrolyzing various precursors (e.g., pitch, glucose) to coat carbon layers on the pre-prepared silicon nanoparticles [17–21].

Cui et al. designed a hierarchical pomegranate-structured Si@C composite and a nonfilling carbon-coated porous silicon micro particle via the pyrolysis of resorcinol–formaldehyde resin (RF), respectively [23, 82]. And Yu et al. prepared double carbon shells coated Si nanoparticles via chemical vapor deposition (CVD), with acetylene as carbon source [24]. All these designs provide sufficient voids to allow large volume changes of Si during the lithiation/delithiation. However, most of Si@C composites were prepared in separate steps by either pre-coating or post coating carbon on silicon nanomaterials. It led to a complicated preparation strategy.

Continued interest in high performance lithium-ion batteries has driven the development of new electrode materials and their synthesis techniques, often targeting scalable production of high quality nanoceramics (< 100 nm in diameter), which may offer performance improvements. However, there are a number of hurdles, which need to be overcome to move away from current batch synthesis methods that offer poor reproducibility or lack of control over crystallite attributes, particularly at larger scale syntheses. Continuous hydrothermal flow synthesis (CHFS) processes are a promising route for the direct and controlled manufacture of Li-ion battery electrode nanoceramics. Such processes use superheated water and metal salt mixtures as reagents. In a typical CHFS reaction, a feed of supercritical water (above the critical point of water (TC = 374 °C and Pc = 22.1 MPa), is rapidly mixed in an engineered mixer [1] with a metal salt/base aqueous precursor feed (at ambient temperature and the same pressure), resulting in rapid formation of the corresponding nanocrystallite oxide in the water. This nucleation dominated reaction occurs as a result of the metal salts being supersaturated upon mixing with sc-water and also instantly being hydrolysed and dehydrated under these exotic reaction conditions. The nascent nanocrystallite metal oxide stream in water is then cooled in process and then can be constantly collected from the exit of the CHFS process as an aqueous nanoparticle slurry at ambient temperature. The cleaned crystallites (e.g. via dialysis) can be obtained as a wet solid and then freeze-dried to retain maximum surface area and reduce agglomeration. Compared to batch hydrothermal syntheses, CHFS type processes typically produce very small nanoparticles (< 10 nm) with a narrow size distribution [2–4]. Additionally, CHFS processes are highly scalable (> 1 kg per hour in the lab of the UCL authors [5]) and can be used to make high quality nanoparticles at scale, with little or no significant variation between those made on the smaller CHFS laboratory scale process.

**Fig. 28:**
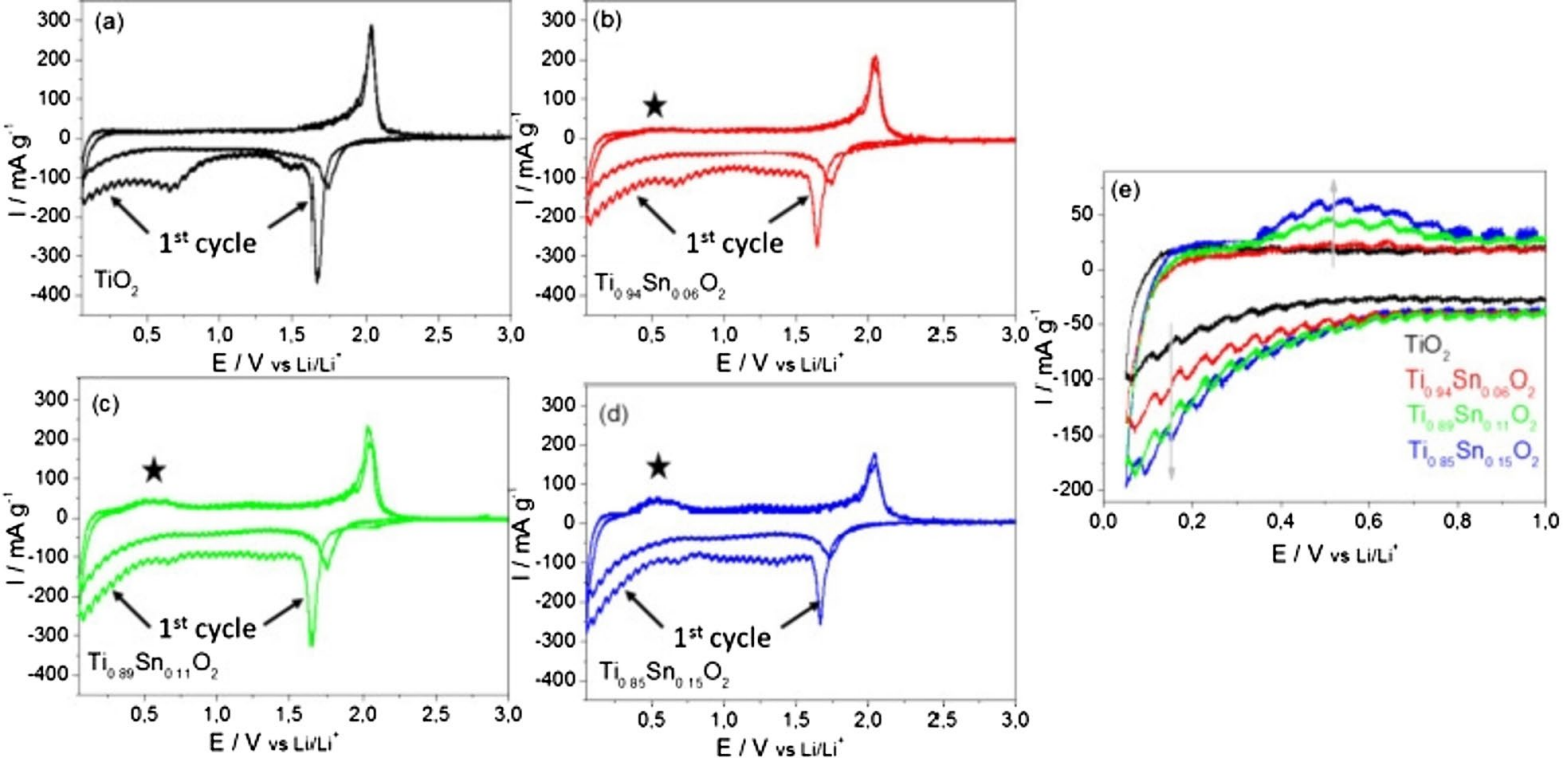
Cyclic voltammograms for the 1st and 2nd cycles for the as-prepared Nano-powder in the potential range of 0.05 and 3 V versus Li/Li^+^ for an applied scan rate of 0.05 mV s^−1^ for **a** undoped anatase TiO_2_, **b** Ti_0.94_Sn_0.06_O_2_, **c** Ti_0.89_Sn_0.11_O_2_, and **d** Ti_0.85_Sn_0.15_O_2_. **e** Specific current versus potential of the 2nd cycle for all materials at lower potentials. The specific current was calculated by taking into account the active material mass loadings [81]

Cyclic voltammetry (CV) measurements at a scan rate of 0.05 mV s^−1^ in the range of 0.05–3 V versus Li/Li^+^, are presented in Fig. 28. A pair of cathodic and anodic peaks were observed in the potential range 1.5 and 2.3 V versus Li/Li^+^, relating to Li-ion insertion into and extraction from the interstitial octahedral site of TiO_2_ (see equation) [81]. Under normal circumstances, a two-phase reaction is expected to occur during lithiation with phase equilibrium of the Li-poor Li_0.01_TiO_2_ (tetragonal) phase and the Li-rich Li_0.55_TiO_2_ (orthorhombic) phase [19, 20]. The detected specific current peak decreased with higher amount of Sn, thereby reducing the amount of pure TiO_2_. The pure TiO_2_ sample showed virtually no electrochemical activity in the potential range between 1.3 and 1 V versus Li/Li^+^ during the first cycle. The increasing specific current during the first cycle between 1 and 0.05 V versus Li/Li^+^, is attributed to solid electrolyte interface (SEI) formation (electrolyte destruction) at lower potentials [13]. There was also likely to be substantive SEI formation at the crystallite surfaces of the Sn-doped materials compared to the undoped TiO_2_, as there was significant electrochemical activity in the range of 1.3 to 1 V versus Li/Li^+^ for the former. However, as the surface area decreases with higher Sn-loading, the initial capacity loss due to the SEI formation may be expected to decrease. The general trend in fact showed that with higher Sn-loading, the initial irreversible capacity loss increased (from 363 mAh g^−1^ for the pure TiO_2_ and 467 mAh g^−1^ for Ti_0.85_Sn_0.15_O_2_).

## Conclusion

1. This article discusses silicon anodes as a representative of alloy anodes. It was observed that the only solution to solve the shredding problem is to use nanotechnology. In this paper, the importance of nanomaterial synthesis was expressed. In summary, how to use nanomaterials with different morphologies to solve the problem and improve power. Although various morphologies were discussed, there was no discussion of structural engineering and the use of carbon conductive materials, which will be discussed in future articles. This was one of the methods of establishing electrical bonding for nanoparticles. There are various structures to prevent the nanoparticles from breaking, the art of which is to create different geometries and the method of their preparation.
2. This article discusses SEI, which is one of the most important topics in most anodes and some high voltage cathodes. This article discussed the problem of alloy anode fragmentation, while which is due to the continuous growth of SEI. It turned out that in order to have a proper cycle life, this problem must be overcome. According to the given examples, using a suitable design at the nanoscale, in addition to providing free volume change of silicon, this volume change does not occur in contact with the electrolyte.
3. The carbon coating on the anode can increase the conductivity from 13–110 to 2.05 S/cm. Doping can enhance performance by increasing the conductivity of electrons and even ions and providing more space within the network along with Nano sizing, which may be appropriate for new projects, which is more a Nano-topic in Nano synthesis than how it accompanies matter. Synthesize with Nano-dimensional doping until there is a discussion about the effect of Nano on improving anode performance. This article discusses titanium oxide anodes, which is one of the most commercially important anodes. It was found that nanotechnology greatly improves the performance of these anodes. Nano sizing has also been shown to affect even the electrochemical and chemical-physical nature (such as charge–discharge curve deformation and greater capacity in surface layers).
4. In this paper, exchange anodes are introduced and their complex operation is described. It was found that many problems, such as alloy anodes, can be solved by Nano damaging the active material. The special effects of Nano were expressed as a change in mechanism.

## Data Availability

All data generated or analyzed during this study are included in this published article.
